# Bone aerophones from Eynan-Mallaha (Israel) indicate imitation of raptor calls by the last hunter-gatherers in the Levant

**DOI:** 10.1038/s41598-023-35700-9

**Published:** 2023-06-09

**Authors:** Laurent Davin, José-Miguel Tejero, Tal Simmons, Dana Shaham, Aurélia Borvon, Olivier Tourny, Anne Bridault, Rivka Rabinovich, Marion Sindel, Hamudi Khalaily, François Valla

**Affiliations:** 1 Institute of Archaeology, The Hebrew University of Jerusalem, Jerusalem, Israel; 2 CNRS, UAR 3132 Centre de Recherche français à Jérusalem (CRFJ), Jerusalem, Israel; 3 CNRS, UMR 8068 Technologie et Ethnologie des Mondes PréhistoriqueS (TEMPS), Nanterre, France; 4 Department of Evolutionary Anthropology, University of Vienna, Vienna, Austria; 5 Human Evolution and Archeological Sciences (HEAS), University of Vienna, Vienna, Austria; 6 Seminari d’Estudis I Recerques Prehistoriques (SERP), University of Barcelona, Barcelona, Spain; 7 Department of Forensic Science, Virginia Commonwealth University, Richmond, VA USA; 8 CNRS, UMR 7041 Archéologies Environnementales, Nanterre, France; 9 Laboratoire d’Anatomie Comparée, École Nationale Vétérinaire, Agroalimentaire et de l’Alimentation (ONIRIS), Nantes, France; 10 CNRS, UMR 7307 Institut d’ethnologie mediterraneenne, europeenne et Comparative (IDEMEC), Aix-en-Provence, France; 11 Institute of Earth Sciences, National Natural History Collections, The Hebrew University of Jerusalem, Jerusalem, Israel; 12 Israel Antiquities Authority (IAA), Jerusalem, Israel

**Keywords:** Anthropology, Archaeology

## Abstract

Direct evidence for Palaeolithic sound-making instruments is relatively rare, with only a few examples recorded from Upper Palaeolithic contexts, particularly in European cultures. However, theoretical considerations suggest that such artefacts have existed elsewhere in the world. Nevertheless, evidence for sound production is tenuous in the prehistoric archaeological record of the Levant, the study of music and its evolution being sparsely explored. Here we report new evidence for Palaeolithic sound-making instruments from the Levant with the discovery of seven aerophones made of perforated bird bones in the Final Natufian site of Eynan-Mallaha, Northern Israel. Through technological, use-wear, taphonomic, experimental and acoustical analyses, we demonstrate that these objects were intentionally manufactured more than 12,000 years ago to produce a range of sounds similar to raptor calls and whose purposes could be at the crossroads of communication, attracting hunting prey and music-making. Although similar aerophones are documented in later archaeological cultures, such artificial bird sounds were yet to be reported from Palaeolithic context. Therefore, the discovery from Eynan-Mallaha contributes new evidence for a distinctive sound-making instrument in the Palaeolithic. Through a combined multidisciplinary approach, our study provides important new data regarding the antiquity and development of the variety of sound-making instruments in the Palaeolithic at large and particularly at the dawn of the Neolithic in the Levant.

## Introduction

The Natufian archaeological culture (c. 15,000–11,700 BP) marks the transition from hunter-gatherer Palaeolithic societies into fully-fledged agricultural economies of the Neolithic^1^. The Natufians were the first hunter-gatherers in the Levant to adopt a sedentary lifestyle, a dramatic economic and societal change associated with growing social complexity as reflected in various aspects of their material culture (e.g., graveyards, artistic manifestations, and durable stone-built structures)^2^.

**Figure 1:**
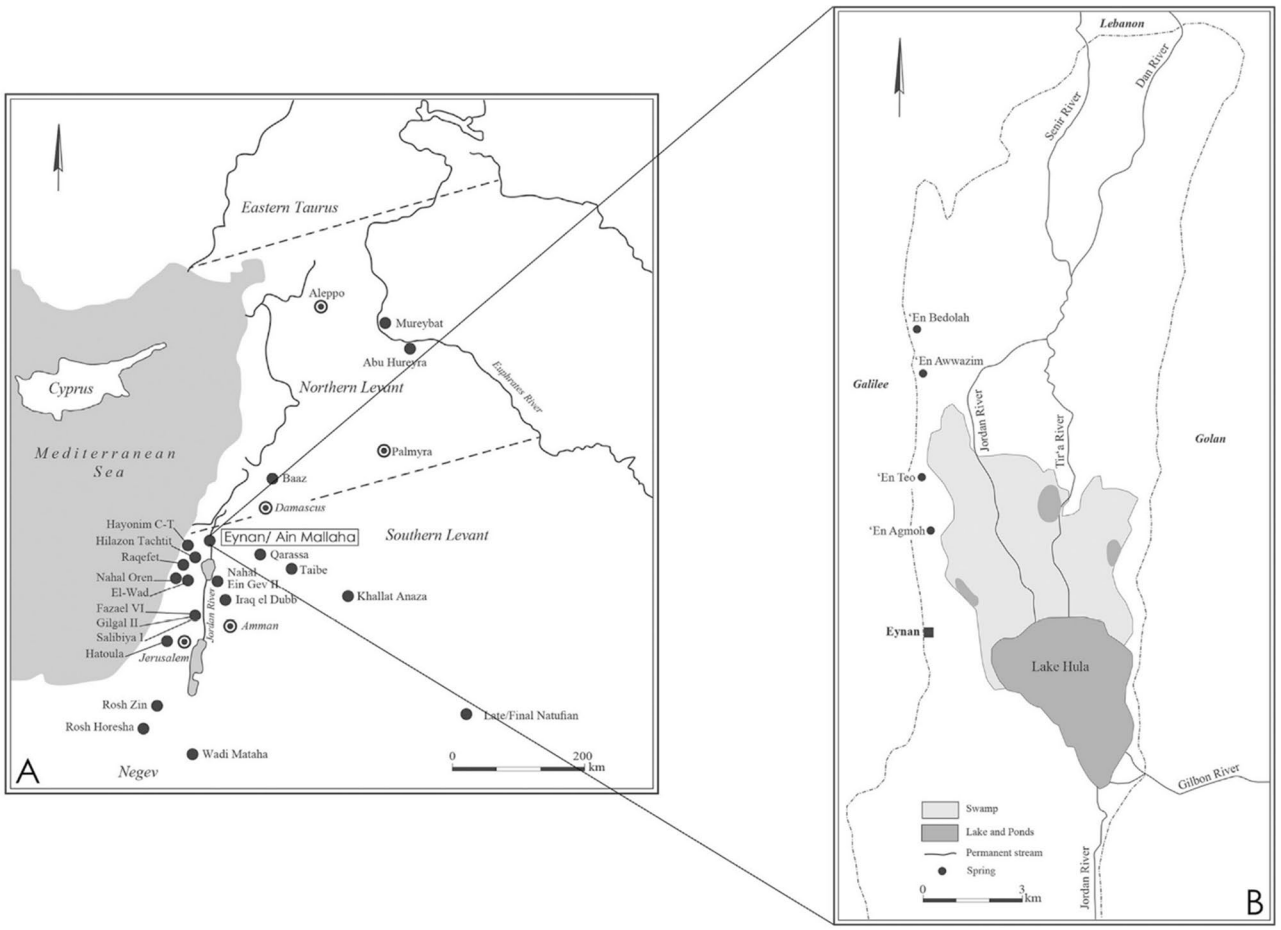
(**A**) Map of the distribution of Late/Final Natufian sites in the Levant; (**B**) Hydrographic Map of the Hula Basin (CAD A.B. and H.K.).

Excavations at Eynan-Mallaha (1996–2005) by F. R. Valla and H. Khalaily^3–6^ yielded over 1112 bird bones^7^ (T.S., *in prep*; Table S1) from the Final Natufian layer (Ib) dated between 10,730 and 9760 cal BC^8^ (Table 1). The site, in the Hula Lake Basin of the Upper Jordan Valley (Fig. 1), is known for its rich Natufian deposits spanning most of the duration of this cultural entity^9–15^. The Final Natufian occupation is circa 50 cm thick, over an excavated surface of about 120 m^2^ and includes five distinctive stone constructions (i.e., shelters) distributed into two temporally separated building phases^16^. Dense concentrations of bird bones are found in dwellings, hearths and graves^7^. The Natufian of Eynan-Mallaha exhibit a clear subsistence preference for the exploitation of wintering waterfowl (75.4% of the Minimum Number of Individuals: MNI) with a distinct but separate emphasis on hunting of birds of prey (13.4% of the MNI) for their talons (terminal pedal phalanges)^5^, which might have been used as tools^17,18^ or for ornamentation as seen in earlier Palaeolithic cultures^19–21^. All other species of birds were of secondary importance and hunted opportunistically.

**Table 1:** List of AMS dates of the level Ib (Final Natufian) at Eynan-Mallaha. The results were calibrated based on Intcal13 ^14^C calibration data set and calculated by calib 7.0.4 program^6^.

Ref. samples	Layer/struct	Ref. lab	Age BP	SD	Cal. BC 1 $\upsigma$ (IntCal13)	Cal. BC 2 $\upsigma$ (IntCal13)
EM05 K95b 1031–2	Ib/St.230	GifA 70,013	10,200	50	10,061–9858	10,157–9760
EM97 R97 6165	Ib2/St.215	GifA 99,332	10,530	100	10,705–10,433; 10,319–10,294	10,742–10,170
EM99 R98c 7657	Ib2/St.228	GifA 100,400	10,540	90	10,696–10,448	10,743–10,272; 10,268–10,196
	Average Layer Ib2				10,639–10,460	10,732–10,285

**Figure 2:**
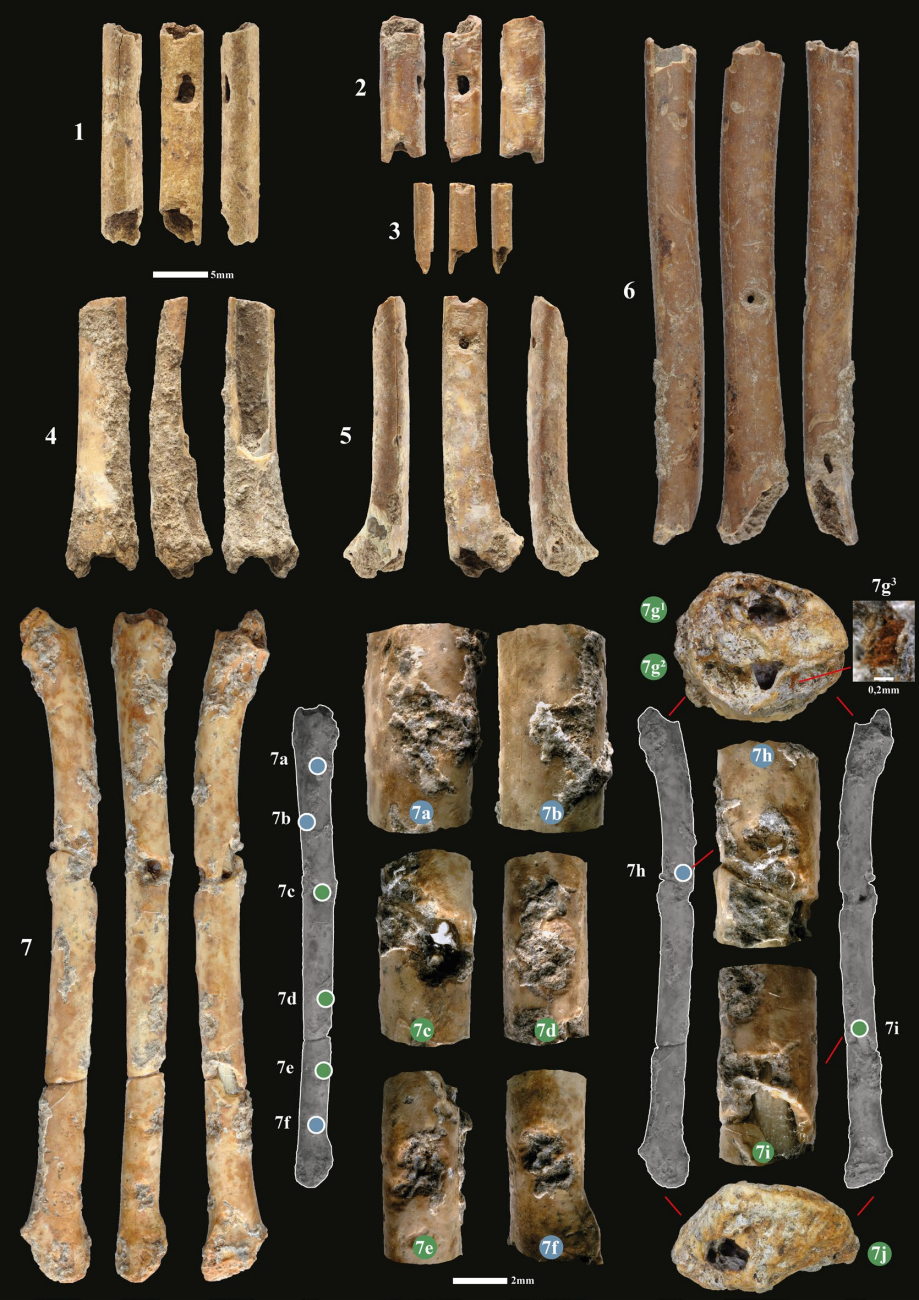
Bone aerophones from Eynan-Mallaha, level Ib (Final Natufian). 1: EM99 7201; 2: EM96 5564; 3: EM97 6182; 4: EM99 7414; 5: EM04 9363; 6: EM98 6581; 7: EM98 7026 with details of the 10 worked areas: 7c/7d/7e/7i being finger-holes (in green), 7a/7b/7f./7 h being shallow notches (in blue), 7 g the mouthpiece with two perforations (7g^1^ and 7g^2^) and a residue of colouring material (7g^3^), 7j the distal end. (CAD and photos L.D.).

A re-evaluation of all the avifauna remains from the Final Natufian of Eynan-Mallaha produced new evidence for distinctive sound-making instruments of a kind never identified before in the wider Palaeolithic record. We identified one complete and six fragments of worked bone as aerophones (Table 2; Fig. 2; Fig. S1-8). The majority of the few Palaeolithic sound-making instruments known today come from Europe^22,23^. The oldest, dated to 40,000 years ago, are the Early Upper Palaeolithic (Aurignacian) bird-bone and mammoth-ivory aerophones from the Swabian Jura in southwestern Germany^24^. To this date, no sound-making instruments were recognized in the Levantine archaeological record from previous Palaeolithic cultures, nor from the later, Neolithic cultures. Sound production in the Natufian of the Levant has previously been suggested, with only a few studies hinting at possible media/instruments for such a practice: a “belt” of bone pendants interpreted as strung rattles^25,26^, fragments of worked bone objects interpreted as bullroarers^26,27^, a fragment of a worked vulture ulna interpreted as a flute without holes^27^ or even the supposed sound of pounding boulder mortars involved in funerary practices of Late Natufian contexts^28^. Altogether, sound-making instruments in the prehistoric Levantine archaeological record are yet to be thoroughly explored, indicating the great potential for further research. Thus, the multidisciplinary methodology developed in this article serves as the first systematic attempt to approach the issue.

**Table 2:** List of the bone aerophones from Eynan-Mallaha, level Ib (Final Natufian).

No on figures	Excavation catalogue	Square	Structure	Taxon	Element	Side	Portion	Shaft perforation (Nbr)	Face perforated (Nbr)	Notch or incisions (Nbr)	Length (mm)	Width shaft (mm)
1	EM99 7201	K95a	Stony layer	*Fulica. Atra*	ulna	R	Shaft	≥ 2	1	–	20.34	3.86
2	EM96 5564	K96b	Loc.210	*Anas sp.*	ulna	L	Shaft	≥ 2	2	2	13.57	4.07
3	EM97 6182	J96c	Loc.203	*Anas sp.*	Radius	L	Shaft	≥ 1	1	–	8.31	2.6
4	EM99 7414	K95d	Stony layer	*Anas sp.*	ulna	R	Proximal + Shaft	≥ 1	1	–	25.27	4.64
5	EM04 9363	I91b	Loc.200	*Anas crecca*	ulna	L	Distal + Shaft	≥ 2	1	–	25.68	4.76
6	EM98 6581	S95	Stony layer	*Anas sp.*	Humerus	L	Shaft	≥ 2	2	1	45.68	5.3
7	EM98 7026	H91a	Loc.200	*Fulica. Atra*	ulna	L	Complete	4	2	4	63.42	4.1

**Figure 3:**
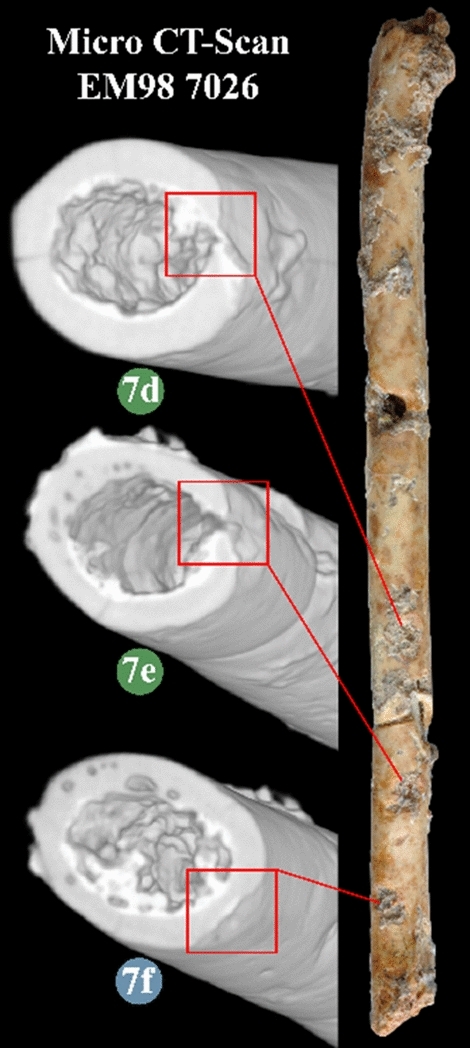
Mico CT-Scan cross-section of three worked areas covered with concretion on the complete aerophone (EM98 7026) showing that 7d and 7e are perforations and 7f. a shallow notch. (CAD and photos L.D.)*.*

The aerophones from Eynan-Mallaha are all made of wing long-bones (one humerus, five ulnae, one radius) whose diaphysis have been perforated one to four times to form finger-holes. In the three cases where the epiphysis is still present, it has also been perforated to form the mouthpiece or the distal end of the object. To these finger-holes are added markings on three bones, either notches (Fig. 2.7) or a series of small parallel incisions located near the finger-holes (Fig. 4.2, 4.6), which are potentially linked to the placements of the fingers on the instrument. All the worked areas show contact-wear traces indicating that all instruments have been used. When looking at the state of preservation, most of the fractures are old, but some were caused by the excavation process. It is the case of the complete aerophone (Fig. 2.7), which was broken in three pieces when discovered in 1998 and glued shortly thereafter, as were several other broken bird bones. Most of the remains are unburnt bones, although the shade of the humerus aerophone (Fig. 2.6) indicates that it may represent a heated bone. As several bones and their technical traces are partially covered by encrustation, the micro-CT-Scan helped us to overcome the difficulty of analysing the bone surfaces (Fig. 3). Attributing the perforated bones from Eynan-Mallaha to acoustic instruments is based on the criteria developed by F. d'Errico and G. Lawson^29^, among them: (1) feasibility, (2) ethnographical parallels, (3) ancient documentary support (text or image), (4) contemporary archaeological support, and (5) efficiency. It is also based on an extensive archaeological and ethnographical corpus of such devices from diverse periods and regions^22,24,30–34^.

## Results

The makers of the aerophones carved the instruments from bird bones identified as the Eurasian teal (*Anas crecca*) and the Eurasian coot (*Fulica atra*). Both species are represented equally in the avifauna assemblage (respectively 11.2% and 10.7% of the Number of Identified Specimens: NISP)^5^ (T.S., *in prep*). Four aerophones have been identified only at the level of *Anas sp.* (surface-feeding ducks) and are in the size range of several small ducks identified at the site (e.g., Garganey: *A. querquedula*, Pintail: *A. acuta*, Shoveler: *Spatula clypeata*). These small ducks are all winter migrants to the Levant and together represent 17.3% (n = 193) of the total NISP. The preferential selection of bird wing bones with their empty diaphysis, offering a long air pipe, for making aerophones corresponds to a universal trend in sound-making instruments as seen in the archaeological and ethnographical record^22,23,30,35^. Nevertheless, the choice to use the bones of small birds raises questions. Indeed, the Natufians from Eynan-Mallaha hunted other, larger species, such as birds of prey (*Accipitridae*), larger waterfowl (Goose, Swans) and especially the Mallard (*Anas platyrhynchos*), which alone represents 22.5% (n = 250) of the total NISP. Thus, selecting short and narrow bird bones as blanks for wind instruments appears to be more of a deliberate choice rather than a constraint of availability. Given that the length and diameter of the bird bone influence the sound production^36^ it seems that the choice is less about the species used than the sound produced by these bones whose air pipe is smaller. This choice is not without consequence, as experiments have shown that the narrower the diameter of the bone is, the more difficult it is to play^36,37^.

These parameters also indicate that, contrary to what is observed in different archaeological cultures of the European Upper Palaeolithic (ulna in Isturitz and radius in Geissenklösterle^36^), the fact that the Eynan-Mallaha Natufians used different wing bones (humerus, ulna and radius) to make aerophones probably reveals the search for varied sound production. This assumption will be tested with further experimental replicas made with humerus and radius bones. The small size of these instruments raises other questions. Indeed, if one considers that their size, in their functional state, corresponds at most to that of the complete aerophone (length of 63.4 mm and a diameter of about 4 mm (Fig. 2.7)), the mastery of these instruments must have required a certain period of training and a level of dexterity. The short distances between the finger-holes of certain aerophones (Fig. 2.1;2.5; Figs. S1; S5; S9) (e.g., 2 mm from 1a to 1b or 5a to 5b) also requires a certain agility. Training with the experimental replicas and comparison with similar-sized bone aerophones from ethnographic contexts^38^ will allow us to expand on this point.

On the three aerophones that conserve at least one epiphysis (Fig. 2.4;2.5;2.7; Table S2), the extremities of the bones have been perforated by pressure (Fig. S11; Fig. S12; Fig. S13) and bear signs of contact use-wear of varying intensity. On the complete ulna aerophone (Fig. 2.7), the proximal epiphysis has been perforated twice (Fig. 2.7 g; Fig. S10) (caudal and cranial views) to form the mouthpiece. Those two perforations are not equal in size (4.8 mm^2^ and 1.9 mm^2^), which might have been done on purpose to influence the airflow, given that the mouthpiece shape conditions the sonority and the way of playing the instrument^22^. The mouthpiece displays a fairly well-developed contact use-wear since it extends further on the diaphysis. According to the typology of aerophones^22^, the mouthpiece allows us to classify the complete aerophone of Eynan-Mallaha as a flute with an unfabricated air duct because the player makes the duct during play with his/her mouth. As it has been demonstrated with the experimental replicas (Audio S1; Fig. S14), the air flow is brought in front of the edge of the perforation (Fig. 2.7g^1^; Fig. S10) which forms the bottom of a notch. Thus, we can classify the Eynan-Mallaha aerophone as a notched flute (like the traditional Quena flute from the Andes), one of the most difficult to play because the player has to hold the instrument in place against his/her lips so that the breath reaches the precise operational point (Fig. S14). According to Hornbostel and Sachs' classification of musical instruments^39^, it could be related to the category of “edge instruments or flutes (narrow stream of air is directed against an edge)”.

On the distal end of the complete instrument, there is a single perforation (Fig. 2.7j) (cranial view), which forms the end of the air column and whose size (3.05 mm^2^) is probably not random. The only other distal end perforation preserved in its original shape (Fig. 2.5; Fig. S9.5c), made on a distal ulna, is twice as large (6.4 mm^2^). Following the same trend, the distance between the last diaphysis perforation and the distal end perforation is shorter on the complete instrument (Fig. 2.7e-j = 11 mm) than on the broken one (Fig. 2.5; Fig. S9.5b-c = 17.7 mm). Experimental replicas have shown that these variations in the size and position of the perforations on the bone influence the sound of the instruments^36^.

**Figure 4:**
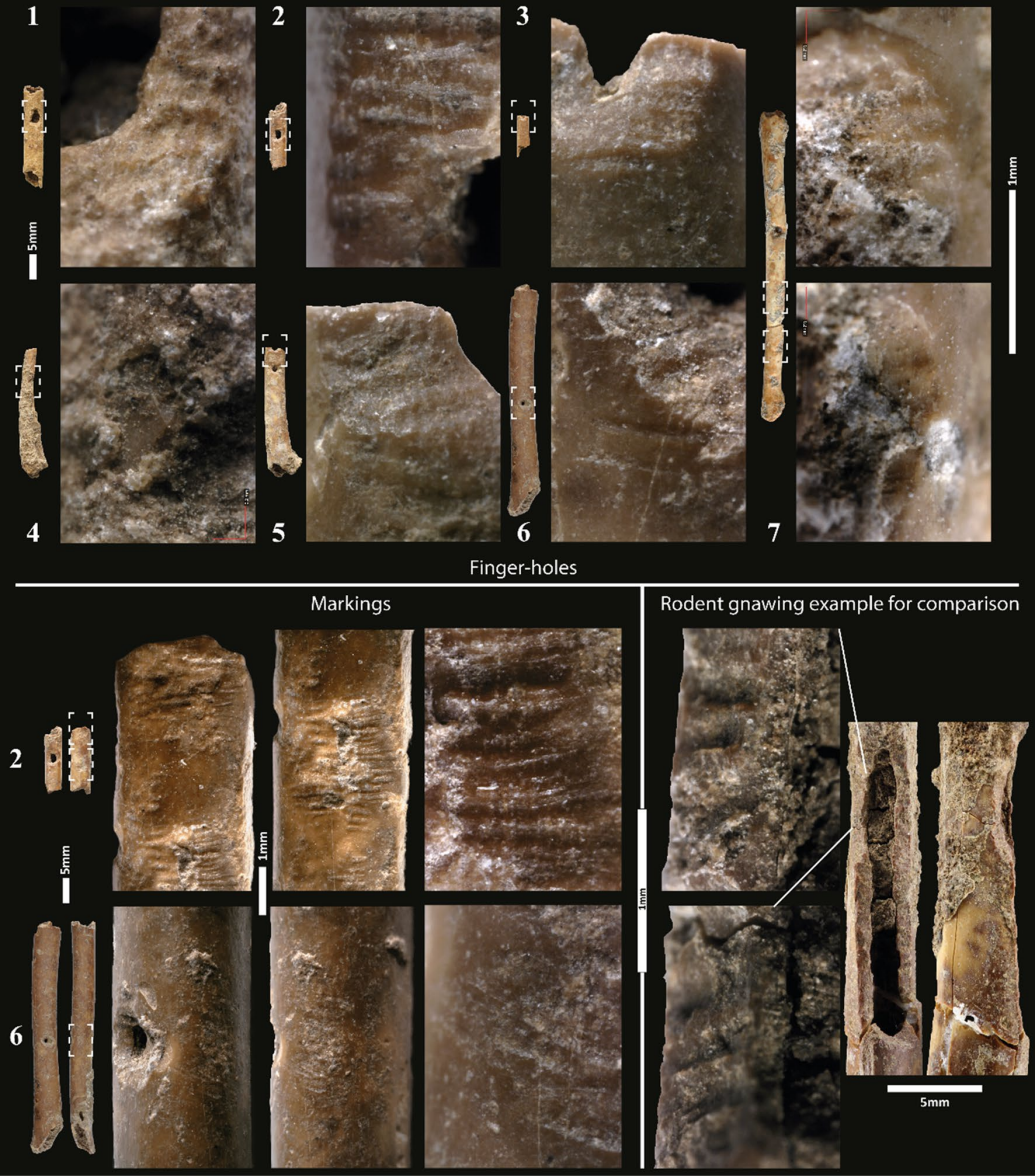
Details of the finger-holes and markings of the bone aerophones from Eynan-Mallaha, level Ib (Final Natufian). 1: EM99 7201; 2: EM96 5564; 3: EM97 6182; 4: EM99 7414; 5: EM04 9363; 6: EM98 6581; 7: EM98 7026 (worked areas 7d and 7e); compared to rodent gnawing on a carpometacarpus of *Anas* sp. (EM01 8477). Note the slope of the gnawing traces towards the outside of the perforation compared to anthropogenic perforations with the grooving traces slope going inside (Magnification 50-250x). (CAD and photos L.D.).

The total 14 perforations on the diaphysis of the 7 aerophones, interpreted as finger-holes (Fig. 2.7; Fig. S9; Table S2), were meticulously made with a short cutting edge (flint burin-bit angle) by transverse and oblique micro-grooving (Fig. 4). The grooving is precise and was obtained by movements alternatively from the right and left edges, reorienting the bone blank several times. The resulting finger-hole margin is in the form of a slightly concave plateau to the fingertip (Fig. 4; Fig. S1-7). This shape serves to improve the pneumatic efficiency of the fingertip seal, which is acoustically essential for the wind instrument to produce the desired output sound tone^30^. The perforation by micro-grooving technique is never used elsewhere in the production of bone tools and ornaments in Eynan-Mallaha, where perforation by rotation is preferred^40–43^. The choice of this unusual technique could result from the physical and technical constraints posed by the small size and fragility of the bird bones used to make the aerophones. Indeed, with small size convex areas to be worked and a compact bone thickness of about 0.5 mm, it might have been challenging to control a perforation by rotation. This hypothesis is confirmed by the experimental replicas and by the aerophone made on a humerus (Fig. 2.6), of which one of the perforations (Fig. S9.6a) was started by grooving and then finished by scraping in rotation, probably owing to a greater thickness of compact bone than on the other wing bones used. In the case of the largest finger-hole of the only complete aerophone (Fig. 2.7c; Fig. S7), the initial perforation, made by transversal grooving, was enlarged and regularised by rotational scraping.

The perforations are aligned either on one face of the bone (Fig. 2.1 and 2.5) or staggered on two faces (Fig. 2.2, 2.6 and 2.7) as seen in other prehistoric bird bone aerophones^34^. Most of the perforations are situated on the convex side of the bone (caudal face of the ulna and humerus) (Fig. 2.4, 2.5, 2.6 and 2.7; Table S2). The finger-holes can be oval and irregular (Fig. 2.1 and 2.2) to circular and regular (Fig. 2.5 and 2.7). This difference in shape is accompanied by a difference in size, the perforations ranging from 0.35 mm^2^ (Fig. S9.6a; Fig. 2.7i) to more than 3 mm^2^ (Fig. S9.1b = 3.1 mm^2^; Fig. 2.7c = 3.3 mm^2^), allowing a ten times bigger air flow. As suggested by Zhang^34^ for Neolithic Chinese bird bone flutes, the smallest perforations could have been made to correct the off-pitch tone of the bigger finger-holes. On the complete aerophone the distances between the finger-holes are varied, but a pattern is recognisable. There are two different spacings with a large finger-hole (Fig. 2.7c) separated, by a large empty space (11.1 mm), from a concentration of 3 smaller finger-holes (Fig. 2.7d,e,i). On three fragmented instruments made on ulna of a similar size (Fig. 2.1, 2.2 and 2.5), we note a regularity in the spacing of the perforations ranging from 2 to 2.8 mm (Table S2), suggesting that the position of the finger-holes may have been predetermined, possibly by use of a template. As the bone size and the number and spacing of the finger-holes influence the sonic output, notably the frequency spectrum^36^, the similarity between them may suggest an attempt to achieve a similar range of sounds by standardising the instruments.

On three of the instruments, markings (Fig. 2.2;2.6;2.7; Fig. S9), either shallow notches made by transverse and oblique grooving (Fig. 2.7) or an oval concentration of small parallel incisions (Fig. 4), can be observed on the side of one of the finger-holes. Most probably, those markings were linked to the placement of the fingers on the instruments. On the complete aerophone, three additional markings, in the form of shallow notches (Fig. 2.7a,b,f), are not related to any finger-holes and might be associated with the movement of the fingers during a sequence of play^24,30^.

The technical traces described leave no doubt that the holes are a product of intentional human activity. Indeed, they are very different in size, shape, and distribution from taphonomic alterations (e.g., carnivore puncture, digestion, rodent gnawing (Fig. 4), roots imprints, insect perforations, etc.) that we can observe on other bird bones in Eynan-Mallaha or even elsewhere within the research literature (see references in the Methods section). Moreover, the Natufian technical choices are similar to those made for the Aurignacian and Gravettian aerophones of Geissenklösterle and Isturitz caves^30,36^ as well as several later examples in Europe^22^, the Americas^31–33^ and China^34^. It is notably the case of the micro-grooving technique used to perforate the finger-holes, their margin in the form of a slightly concave plateau to the fingertip, incised markings near the finger-holes, the choice to perforate the convex side of the bone as well as the pattern of two different spacings with a large finger-hole separated, by a large empty space, from a concentration of three smaller finger-holes.

The use-wear on the outer finger-hole margin (Fig. 4) suggests that all the instruments have been used but at different intensities. Although more experimental references are needed, some use-wear studies on Palaeolithic aerophones^30,44^ seem to demonstrate such assumptions. The only humerus aerophone (Fig. 4.6) showed the least intense wear, while one of the ulna aerophones (Fig. 4.2) exhibited the most intense wear. None of the aerophones from Eynan-Mallaha show incisions commonly interpreted as decorations^22^, but residues of a red colouring matter can be seen on the shaft and mouthpiece of the complete aerophone (Fig. 2.7g^3^). These residues have been characterised as a mix of clay and ochre (hematite) by Scanning Electron Microscopy with Energy Dispersive X-ray Spectroscopy (SEM–EDS) (Fig. S10). They resemble the hundreds of others that we have observed on the Natufian shell and bone beads from Eynan-Mallaha^40^ and, in the corpus of bird remains, this is the only occurrence of a bone-bearing residues of colouring matter, disproving a possible taphonomic origin of the residues. The presence of ochre per se in the archaeological remains and the presence of modified ochre fragments have been considered as part of the symbolic behaviour of prehistoric societies of diverse ages and geographical origins^45–48^. Moreover, the association of red colouring or red patterns with aerophones is also documented in the prehistoric^49^ and ethnographic records^50^.

**Figure 5:**
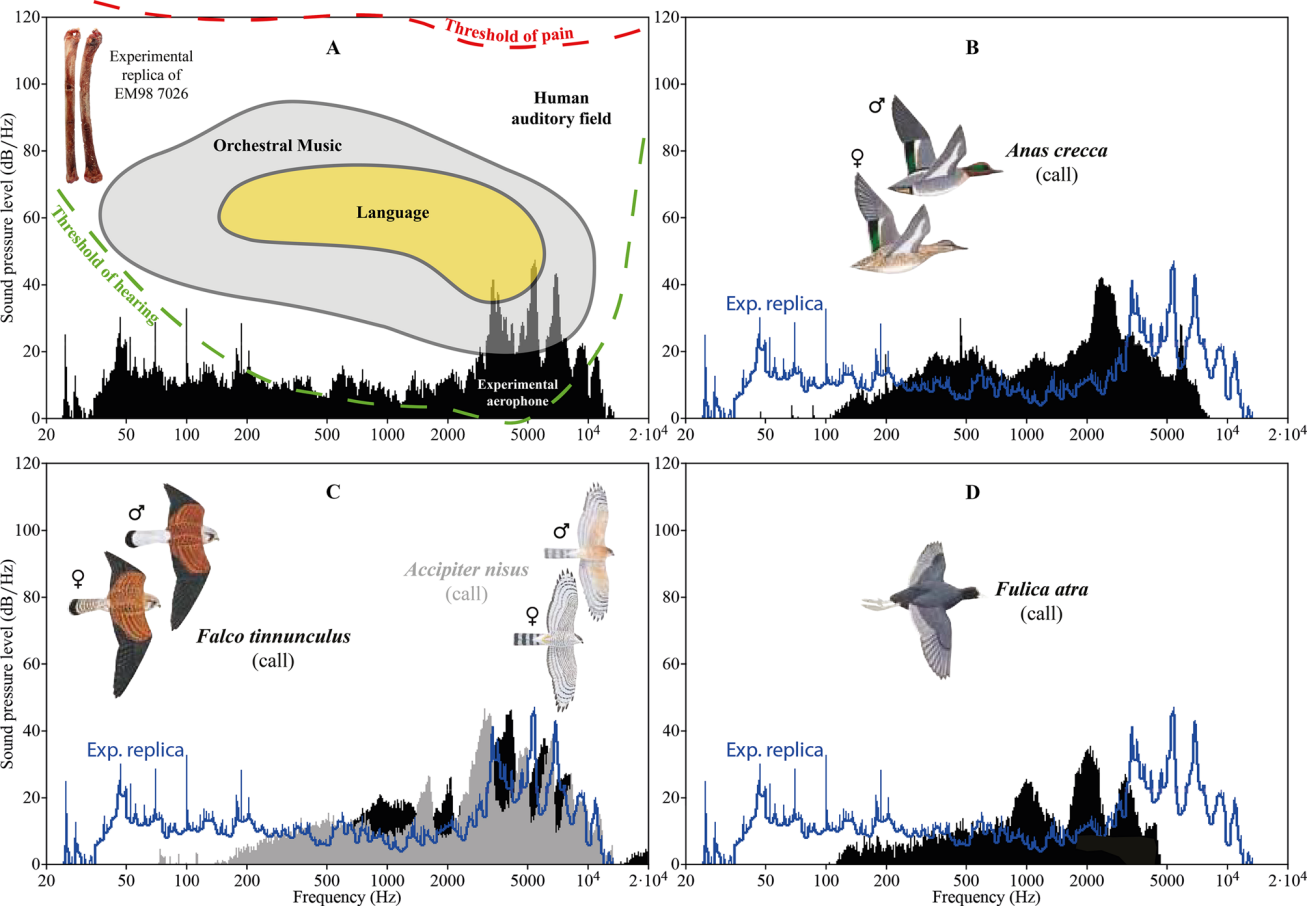
In the human auditory field, spectral analysis of the sounds produced by (**A**) the green bone experimental replica of the complete aerophone EM98 7026 (based on Audio S1); Compared to the experimental replica: (**B**) the Eurasian teal (*Anas crecca*) call (based on Audio S2); (**C**) the Common kestrel (*Falco tinnunculus*) and the Sparrowhawk (*Accipiter nisus*) calls (based on Audio S3 & S4); (**D**) the Eurasian coot (*Fulica atra*) call (based on Audio S5). (CAD L.D.).

**Figure 6:**
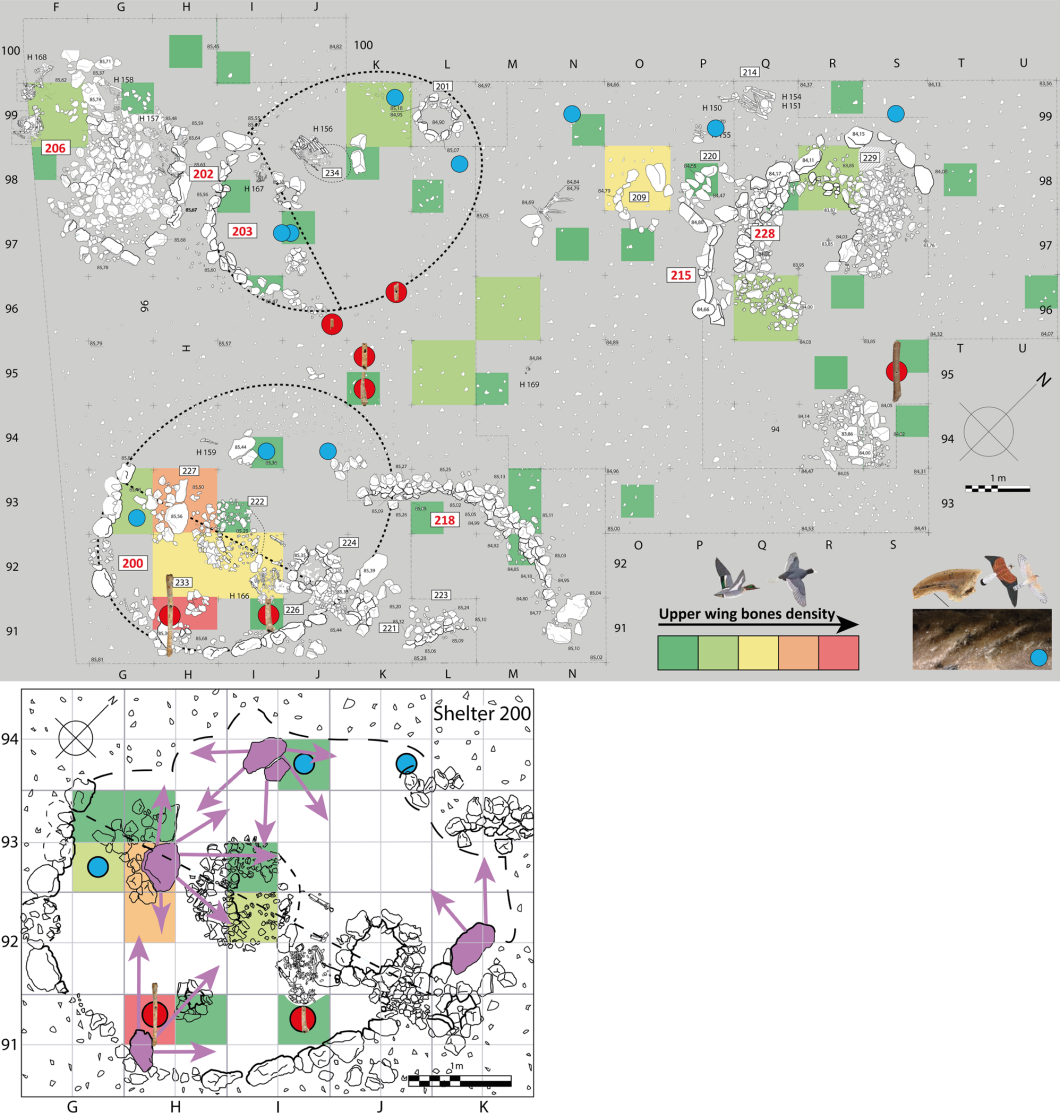
Top: Plan of the layer Ib (Final Natufian) at Eynan-Mallaha (shelters numbers written in red, boundaries indicated for shelters 200 and 203: the south delimited part is the roofed part) with: the position of the 7 bone aerophones indicated by red dots; the spatial distribution (by excavated squares) of upper wing bones (humerus, ulna, radius) of Eurasian teal (*Anas crecca*) and Eurasian coot (*Fulica atra*) (NISP = 63); the position of terminal pedal phalanges (talons) of Common kestrel (*Falco tinnunculus*) and Sparrowhawk (*Accipiter nisus*) indicated by blue dots (NISP = 10). Left: Detail (by sub squares) of Shelter 200 with the representation of the artefact distribution from the identified activity stations (in purple)^14^. (CAD and photos L.D.)*.*

We reproduced experimentally three aerophones similar to the complete one of Eynan-Mallaha (Fig. 2.7). Replicas were made on both green and dry ulna of two female mallard individuals (*Anas platyrhynchos*) (Fig. S11). We used this species, close in size and shape (for female individuals), because of the difficulty in obtaining carcasses of Eurasian coot (*Fulica atra*) used by the Natufians. Our main goals in reproducing the marks observed on the archaeological objects were to assess the technical gestures involved in their production and to demonstrate the possibility of producing melodic (e.g., diverse tonalities and frequencies) sounds with the aerophone. The experimental marks are entirely consistent with those observed on the archaeological aerophones from Eynan-Mallaha. We showed that the Natufian artisans proceeded by pressure on the epiphysis and transversal grooving plus rotational scraping to perforate the finger-holes (Fig. S11; Fig. S12; Fig. S13). We also reproduced several high-quality and high-pitched notes (maximum intensity approximately 65 dBA at 1 m of the instrument) on frequencies and levels in the human auditory field ranging from 2500 to 12,000 Hz (Fig. 5A; Audio S1). We hypothesize that the purposes of the sound produced were to imitate bird calls. In that case, we can see that the three intense high frequencies produced by the replica (3000–4200 Hz, 4400–5600 Hz and 6050–7650 Hz) are not identified in the calls of *Anas crecca* (Fig. 5B) and *Fulica atra* (Fig. 5D) (Fig. S15), the species of which Natufians chose to use the bones to make the aerophones (Table 2). Among the 58 species identified in the Final Natufian of Eynan-Mallaha^7^ (Table S1), the calls of the Common kestrel (*Falco tinnunculus*) and the Sparrowhawk (*Accipiter nisus*) are the only ones that develop similar sound spectra (Fig. 5C; Fig. S16). These two Falconiformes are the most familiar and easily seen raptor. According to the sonic affinity theory^51^, which holds that the action of surrounding sounds shapes the human musical brain, the calls of those familiar raptors should have therefore integrated the acoustic brain of the Natufians. Even if the Common kestrel and the Sparrowhawk have not been discovered in large numbers in Eynan-Mallaha (MNI = 3), they are represented almost exclusively by their talons (NISP = 10 out of 13), some of them bearing anthropic modification traces (LD, *in prep*) (Fig. 6), which indicates their use as personal ornaments and their symbolic status for the Natufians^19–21^. It should also be noted that these two species alone represent the majority (NISP = 10 out of 17) of the raptor talons found in the Final Natufian layer (17 species in total).

We, therefore, believe that the Eynan-Mallaha aerophones were made to reproduce the calls of the valued Common kestrel and Sparrowhawk. This function explains the Natufians' specific choice to use small blanks with reduced air conduct in order to create high-pitched sounds similar to the bird calls. But if the aerophones were functioning as bird calls, what was their purpose? They could have been meant as a decoy used to lure the Common kestrel and Sparrowhawk to facilitate their hunting (i.e., luring birds within shooting distance by imitating their sounds^52,53^) but, with a total of seven aerophones made for only three of those birds identified on the site, the decoy would have lacked effectiveness. In contrast, ethnographic and archaeological evidence from various parts of the world consistently show that in societies where birds by-products (talons, feathers) are used as personal ornaments, vocal and instrumentally made bird imitative sounds have high symbolic value in traditional music and dance^50,54^. For example, during the Sun Dance of the tribes of Plains Indians, a major communal religious ceremony that reflects their relationships with nature, dancers imitate constantly and in unison the call of the symbolically valued Spotted eagle (*Clanga sp*.) by blowing in a whistle made of an eagle ulna bone decorated with feathers and red dots of ochre^50^. For the Kaluli (Great Plateau of Papua New Guinea), rainforest birds are considered to be spirits and their feathers are worn as ornaments. During women’s funerary song weeping and men’s ceremonial poetic songs, bird imitative sounds are intimately associated with the vocal music which allows the bird spirits to speak with the community through the musician^54^. We, therefore, wonder if imitative bird calls were integrated into Natufian musical or dancing practices.

All the aerophones were found discarded with many other forms of occupational wastes of layer Ib (see^14^ for detailed spatial distribution patterns and identified activity stations inside the shelters). The complete aerophone and a fragment also made from an ulna (Fig. 2.5 and 2.7) were found in two adjacent squares within 20 cm depth inside the roofed part of Shelter 200 (Fig. 6). The majority of the remains of *Anas crecca* and *Fulica atra* as the upper wing bones (humerus, ulna, radius) (Fig. 6) and the pectoral girdle bones (scapula, furculum, sternum and coracoid), associated with the largest meat mass in the avian body, are also concentrated in this structure (Fig. S17). There is another concentration of four instruments (Fig. 2.1,2,3,4) in two squares within 10 cm depth containing the Shelter 203 and the stony layer outside it (Fig. 6). This structure concentrates the upper wing bones of a bigger waterfowl, the Mallard (*Anas platyrhynchos*) (Fig. S17), which are nearly absent of the roofed part of Shelter 200 and have not been chosen to make aerophones. The thin archaeological horizon between these aerophones, inside the two areas of concentration (Shelter 200 and outside of Shelter 203), suggests a possible contextual link between them. Shelter 200 and Shelter 203 are also the contexts where most of the talons of the Common kestrel, the Sparrowhawk (NISP = 7 out of 10) (Fig. 6) and the other diurnal raptor have been found.

These aerophone concentrations suggest that there may have been areas of specialised activity, either for storing, playing or discarding sound-making instruments. This possibility is well illustrated by the complete aerophone discovered in the roofed part of Shelter 200 (square H91a), next to an activity station^14^ where the upper wing bones of the birds chosen to make aerophones seem to have been curated (Fig. 6). The only humerus aerophone (Fig. 2.6) was found isolated, deeper in the slope of the stony layer, east of Shelter 215/228, where many bird bones have been discarded (Fig. 6; Figs. S17–S18).

## Discussion

The seven aerophones from the Final Natufian of Eynan-Mallaha share a specialised “*chaîne opératoire*” characterised by a unique manufacturing technique. Given the discretion of the technical traces on the bones, one could expect that similar instruments, not yet identified as such, are still hidden in the avifauna collections of other Natufian sites. We must also remember that the ethnographic record suggests a vast range of sound-making instruments are made from perishable materials^23^. In any case, we can already note that the seven aerophones of Eynan-Mallaha form the largest assemblage of prehistoric sound-producing instruments in the Levant. In a broader perspective, the complete aerophone of Eynan-Mallaha is one of the very few prehistoric sound-making instruments that has come to us in a complete state.

Following musicological terms, music is sounds and notes in their social context, developed during social activities^55^. While the acoustic action is universal (all existing human societies produce vocal or instrumental music), the form and the means it takes are contingent and varied^22^. From the archaeological perspective, one fundamental problem is distinguishing between an artefact's potential to produce sound and the confirmation that this artefact served that specific purpose^29,55,56^. Hence, the global Palaeolithic record of sound-making instruments is scarce too. The earliest secured examples are aerophones from early Aurignacian contexts in southwestern Germany^24,30,36,57^. These examples fit into the archaeological paradigm of modern behaviour and the biological diversity of hominins in the Palaeolithic. Musical instruments are also recognised in later Upper Palaeolithic corpora in Europe (Gravettian and Magdalenian), including, for example, various aerophones and idiophones^58,59^. However, the evolution of music still needs to be completed.

Theoretical considerations suggest that music evolved through time and space^23,55,60–65^. Music, on a phenomenological level, articulates with various aspects of our being, including brain functioning, behaviour, communication and socio-cultural traits^66–68^. Thus, positive paleo-organology (i.e., the research of ancient sound-making instruments) suggests new venues for future research—the prehistory of sound manipulations within their socio-cultural contexts through time and place. This emergent paradigm may expand our knowledge of various functions of different sounds and their co-evolution with biological (brain), behavioural, and, importantly, socio-cultural transformations in our prehistoric pasts.

Our study of the seven Natufian aerophones show that these instruments, meticulously manufactured, demonstrate the existence of a distinct category of objects that might represent a tradition of sound production in Eynan-Mallaha. These wind instruments employ the musically effective finger-hole principle for generating and organising of different sound pitches—the single-player melodic capability^30^—resulting in complex sound-communication behaviour.

Altogether, technological, use-wear, taphonomic, experimental and acoustical evidence combined with comparative record of ethnographic and archaeological examples suggest a new type of Palaeolithic aerophone, which was not identified before. It is unique in the Palaeolithic record of sound instruments both by the chosen medium—i.e., small-sized bones of waterfowl, as well as the sounds it produces which closely resemble falcon calls. This new discovery contributes to the larger picture of music evolution (music sensu*-lato*) a yet unexplored category of sound-making instruments which have been part of the Palaeolithic acoustic environment, and produced artificial sounds resembling natural ones.

Moreover, here we add crucial evidence for the acoustic phenomenon of sound manipulation from a cultural context, which marks a significant change in the history of humankind—the transition into complex agricultural societies manipulating their vegetal and animal environments and accelerating the emergence of new ways of life.

The Natufian’s manipulations of sounds might have functioned in various aspects of their socio-cultural lifeways, either for hunting, communication or ritualised behaviour^25^. Further exploration of this discovery may develop such questions as—what is the nature of the sound produced? How varied or restricted are they? What can we learn about their perception? And, how do they affect human and animal behaviour? Future research into these specifications, concerning the function, perception and effects of the sounds produced by these bone aerophones will be one of the foci of a more in-depth analysis that we will develop in the future. It is now clear that the evolution of music at the transition to agriculture, which articulated the intensification of socio-cultural complexity, was more branched than we supposed before. Thus, the exploration of Natufian acoustics gives a new perspective on this crucial period in human history.

## Materials and methods

### Archaeological methods

All the material studied and reported herein originated in Eynan-Mallaha’s Final Natufian deposits (Layer Ib) excavated by F. R. Valla and H. Khalaily between 1996 and 2005 (Israel Antiquities Authority permit numbers: G-81/1996, G-53/1997, G-77/1998, G-60/1999, G-51/2000, G-62/2001, G-44/2003, G-20/2004 and G-51/2005). Generally speaking, the excavation was conducted using a unit system of a quarter square meter (0.25 m^2^), 5 cm deep. This system was adapted according to the findings. The undifferentiated top of Layer Ib was excavated using a 1 m^2^ grid, shifting to the quarter square meter grid inside and outside structures as soon as they were identified. The fill of each structure was isolated, introducing ‘natural’ boundaries instead of the artificial limits established by the grid. Nevertheless, the artificial grid subdivisions were respected in each structure to allow spatial distribution of the small finds.

The thickness of the units was also adapted to the stratigraphy, resulting in units much thinner than 5 cm, especially when searching for ‘floors’ and surfaces. As a rule, only exceptional pieces were spatially recorded during excavation in the undifferentiated upper part of Layer Ib, which was crowded with small limestone blocks. Inside the structures, all items larger than 5 cm (bones, stones, lithic artefacts and other finds) were mapped. Sediments were wet-sieved using a 1–2 mm mesh and further sorted in the laboratory.

### Taxonomical and anatomical identification

Each bird bone retrieved has been numbered individually by the catalogue numbers of the excavation. All variables regarding the definition of taxa, element, completeness, and surface modifications were recorded in a database. All the remains are now housed at the National Natural History Collections at the Hebrew University of Jerusalem, Israel (NNHC-HUJ). The taxonomic identification of the bones took place in several phases of research spanning some 20 years. Initial identifications of taxa were conducted using the University of Michigan Museum of Zoology’s avian collection. Later work, from ca. 2003 to this publication, was performed at the National Natural History Collections at The Hebrew University of Jerusalem, Israel (NNHC-HUJ).

### Taphonomic analyses

Each bone was examined under a light microscope (magnification 10–40×) and a Dino-Lite AD-7013MZT digital microscope (magnification 30–250×) for surface modifications caused by abiotic and biotic processes, such as carnivores, raptors, rodents, roots and human predation^69–71^. We have distinguished the technical traces from others, characteristic of gnawing, but also perforations, tearing and depressions caused by insects or the beaks, claws, teeth and talons of scavengers or birds of prey^72–81^. Anthropic perforations were differentiated from natural holes based on five main criteria: (i) anatomical location on the bone; (ii) size and nature of the marks; (iii) mode of manufacture; (iv) shape and regularity of the holes; (v) experimental perforations comparison (see description above and Fig. S11; Fig. S12; Fig. S13).

### Eynan-Mallaha aerophones analyses

The aerophones were analysed microscopically with a stereoscopic Olympus SZX10 microscope (magnification 7,8–78×) and a Dino-Lite AD-7013MZT digital microscope (magnification 30–250×) at the Centre de Recherche Français à Jérusalem. Micro CT scans were conducted with a Nikon Micro CT XT H 225 at the Shmunis family anthropology institute, Dan David Center of Human Evolution and Biohistory Research, Sackler Faculty of Medicine, Tel Aviv University. Scanning Electron Microscopy with Energy Dispersive X-ray Spectroscopy (SEM–EDS) micro analyses were performed on a Cryo High-Resolution Scanning Electron Microscope Apreo 2S at the Center for Nanoscience and Nanotechnology (Unit for Nano Characterization) of the Hebrew University of Jerusalem. CAD (computer-aided design) was done with Adobe Illustrator 2022.

### Experimental replicas

The carcasses of a female mallard (*Anas platyrhynchos*) were provided by the comparative anatomy unit of the National Veterinary School of Nantes—Oniris, which is approved for the use of animals for scientific purposes under the number: C 44 274; certification obtained from the prefecture of Loire Atlantique on February 21, 2020, and still in effect today. The experiment was carried out in compliance with the sanitary standards and the respect for animal welfare. The unretouched flint bladelets used in the experiment are of consistent typology with those from the Final Natufian archaeological record.

We recorded every element and factor (time, number of incisions, the position of the hand, etc.) in a specific file designed for this experiment. Moreover, we graphically recorded all the processes with a Canon 6D camera (180 mm, 60 mm and 35 mm Tamron Macro lenses). The experimental marks were also analyzed microscopically with a stereomicroscope Kiowa SDZ-TR-P completed with a microscope camera Motic Moticam-S6. We conducted spectral analyses of the sounds produced by the replica, and bird calls with the free computer softwares Praat (version 6.2.23) and Spek (version 0.8.2). The bird calls of the 58 species identified in Eynan-Mallaha were sampled from the Sonothèque du Muséum national d'Histoire Naturelle of Paris (https://sonotheque.mnhn.fr/) and Xeno-canto.org powered by the Xeno-canto Foundation and Naturalis Biodiversity Center (https://xeno-canto.org/).

## Supplementary Information

Supplementary Information 1.

Supplementary Information 2.

Supplementary Information 3.

Supplementary Information 4.

Supplementary Information 5.

Supplementary Information 6.

## Data Availability

All data needed to evaluate the conclusions in the paper are present in the paper and/or the Supplementary Materials. Additional data related to this paper may be requested from the corresponding authors.
