# Supplementary material for: Bone aerophones from Eynan-Mallaha (Israel) indicate imitation of raptor calls by the last hunter-gatherers in the Levant
Source: Sci Rep. 2023 Jun 9;13:8709. doi: 10.1038/s41598-023-35700-9 (PMC10256695; doi:10.1038/s41598-023-35700-9)
Supplement: Supplementary file 6 — Supplementary Information 6. [file 41598_2023_35700_MOESM6_ESM.docx]

Supplementary Materials for

**Bone aerophones from Eynan-Mallaha (Israel) indicate imitation of raptor calls by the last hunter-gatherers in the Levant**

Laurent Davin*, José-Miguel Tejero*, Tal Simmons, Dana Shaham, Aurélia Borvon, Olivier Tourny, Anne Bridault, Rivka Rabinovich, Marion Sindel, Hamudi Khalaily, François Valla

*Corresponding author. Email: laurent.davin.etu@gmail.com; jose.miguel.tejero@univie.ac.at

**This PDF file includes:**

Figs. S1 to S18

Table S1 to S2

**Other Supplementary Materials for this manuscript include the following:**

Legends for Audio S1 to S5


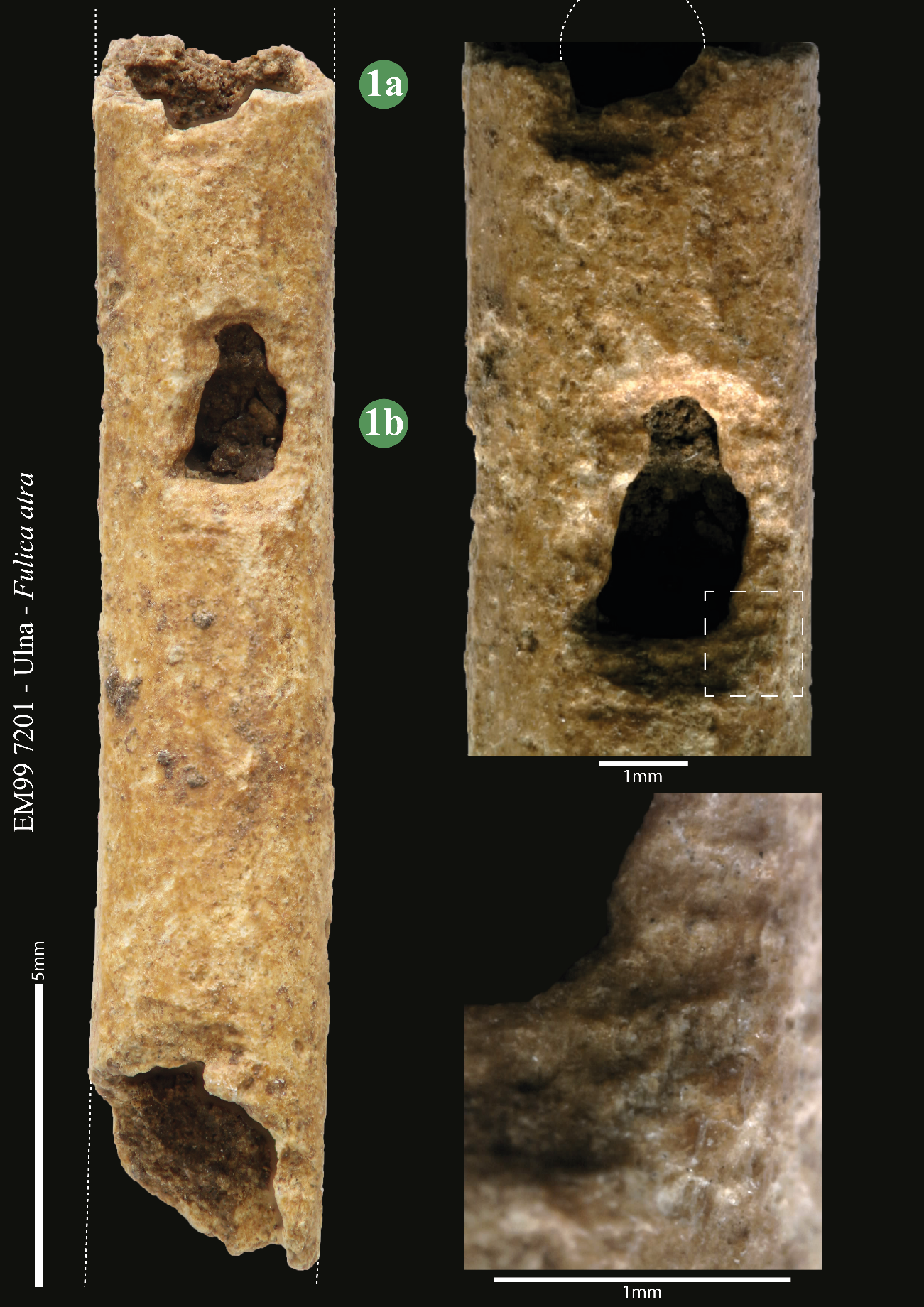


**Fig. S1.** Detail pictures (50-250x) of the technical traces (in green perforations) on the fragmented aerophone (EM99 7201) made of a right ulna of Eurasian coot (*Fulica atra*)*.* (CAD & photos L.D.).


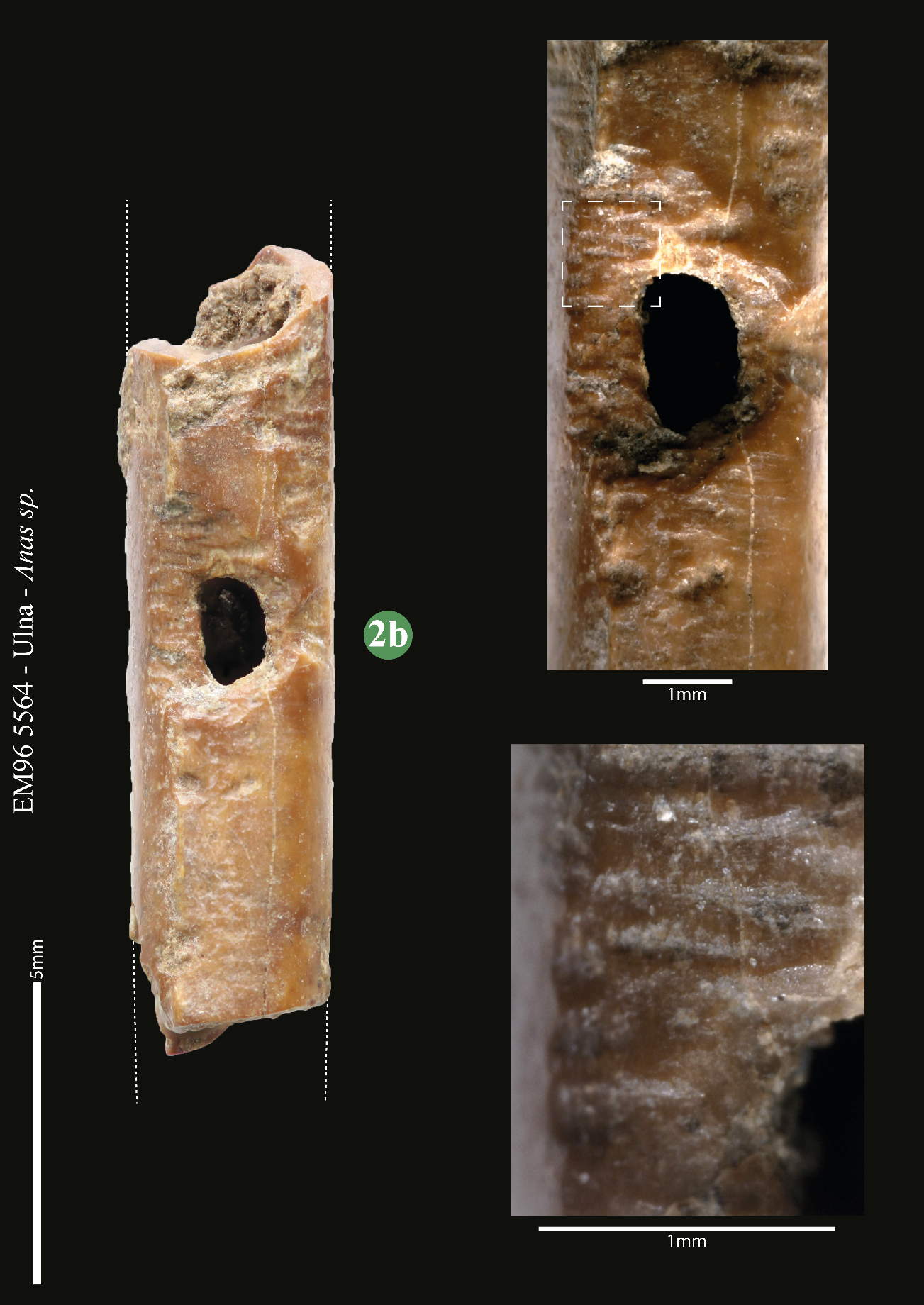


**Fig. S2.** Detail pictures (50-250x) of the technical traces (in green perforation) on the fragmented aerophone (EM96 5564) made of a left ulna of *Anas sp.* (CAD & photos L.D.).


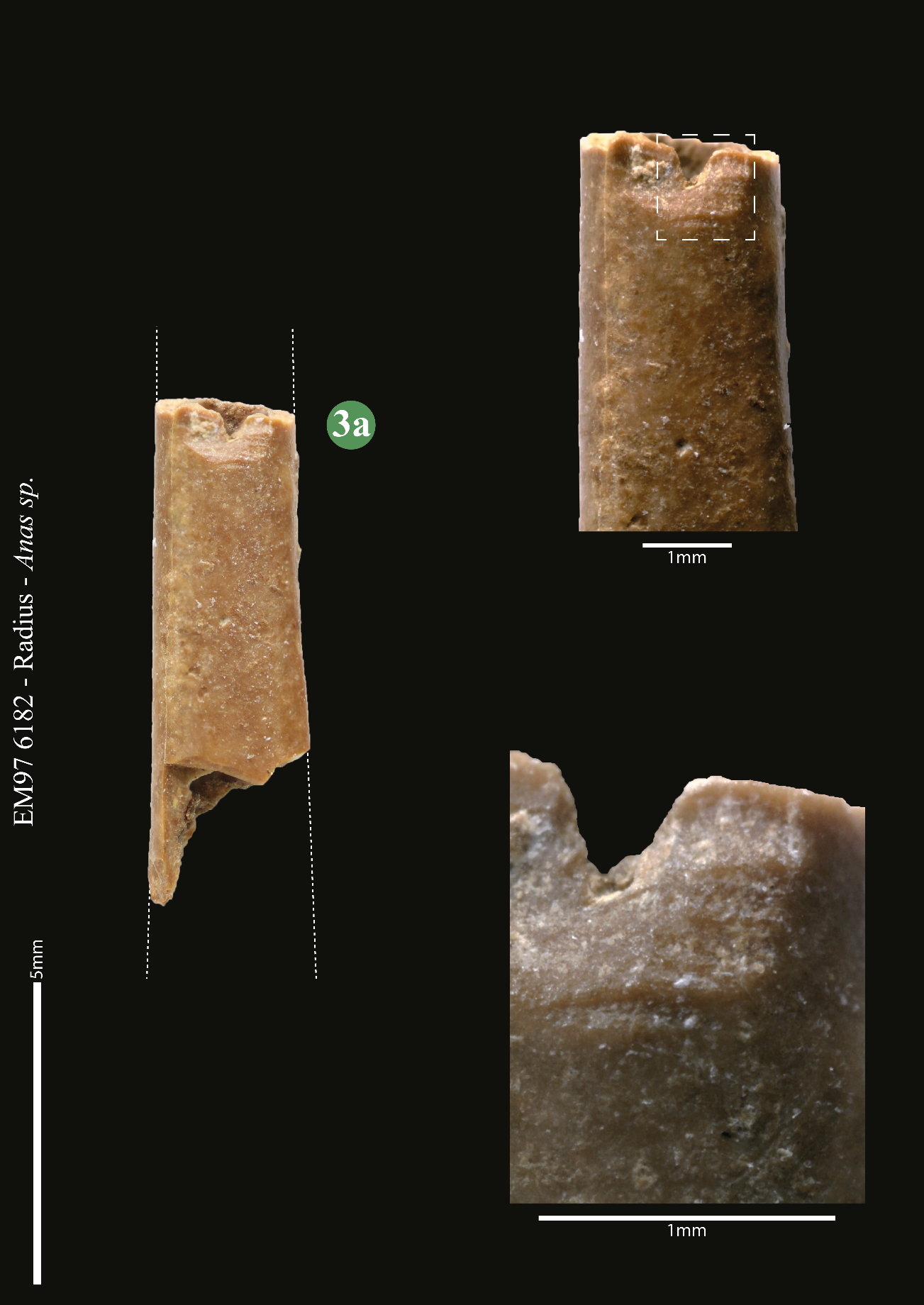


**Fig. S3.** Detail pictures (50-250x) of the technical traces (in green perforation) on the fragmented aerophone (EM97 6182) made of a left radius of *Anas sp.* (CAD & photos L.D.).


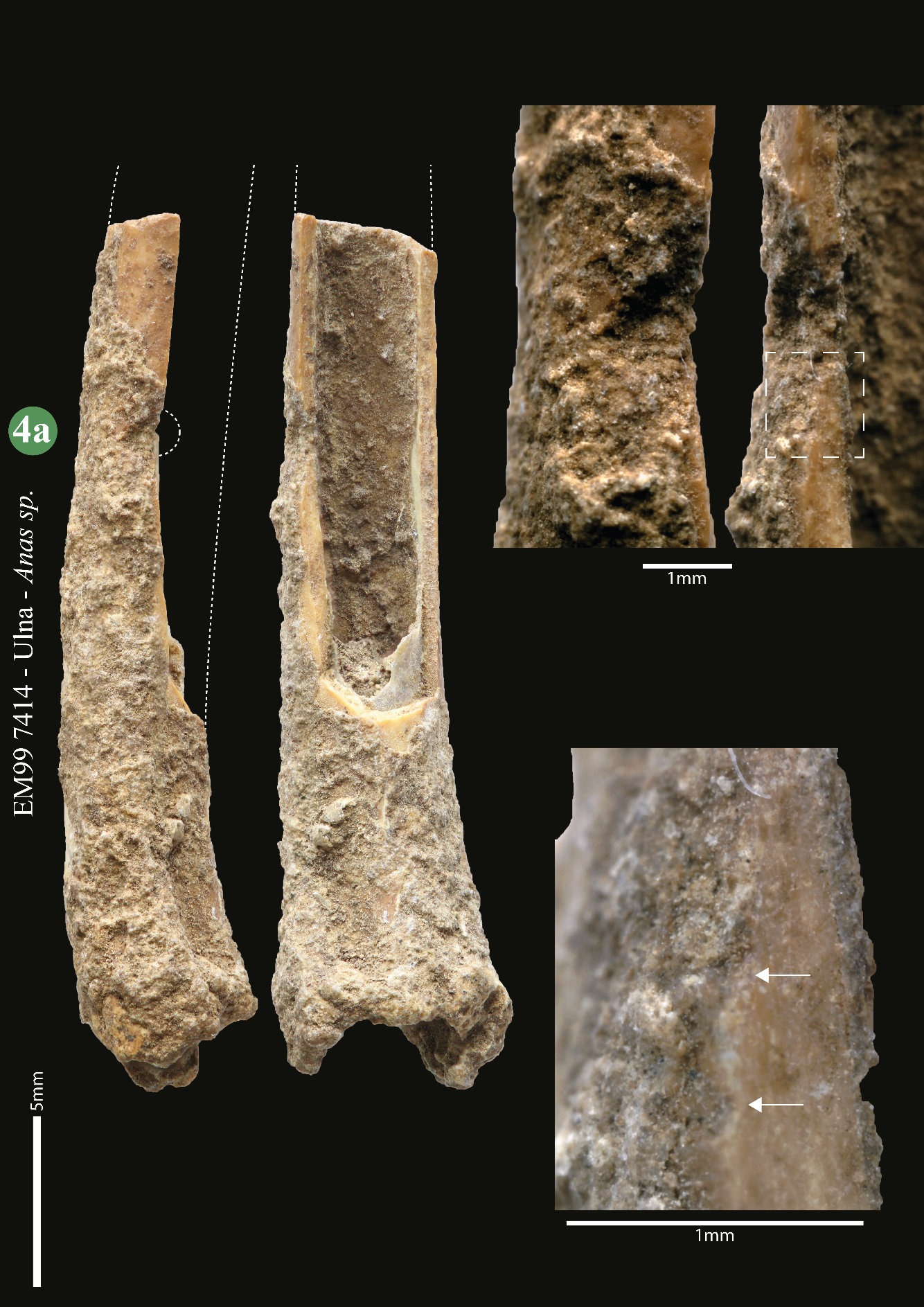


**Fig. S4.** Detail pictures (50-250x) of the technical traces (in green perforation) on the fragmented aerophone (EM99 7414) made of a right ulna of *Anas sp.* (CAD & photos L.D.).


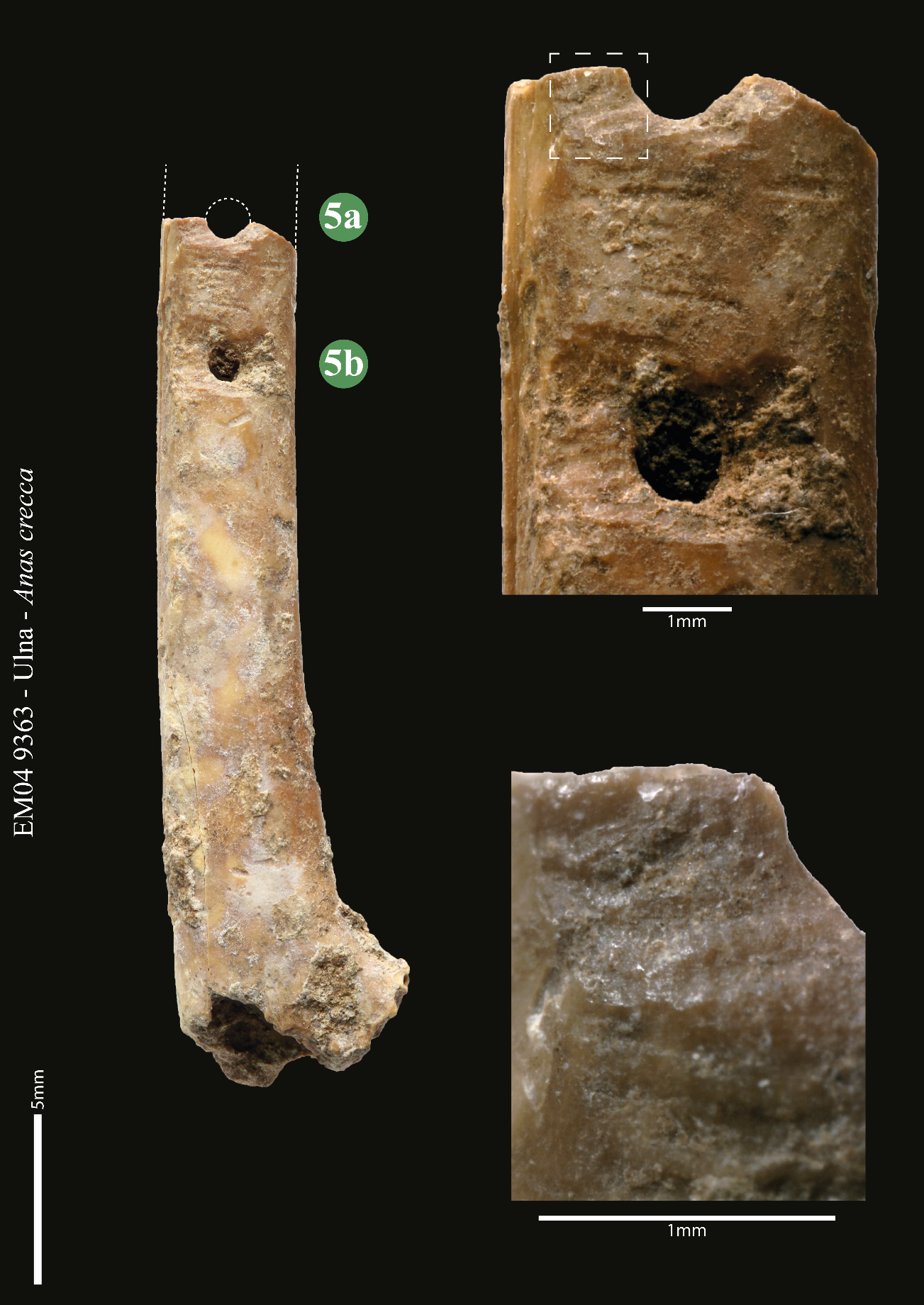


**Fig. S5.** Detail pictures (50-250x) of the technical traces (in green perforations) on the fragmented aerophone (EM04 9363) made of a left ulna of *Eurasian teal* (*Anas crecca*). (CAD & photos L.D.).


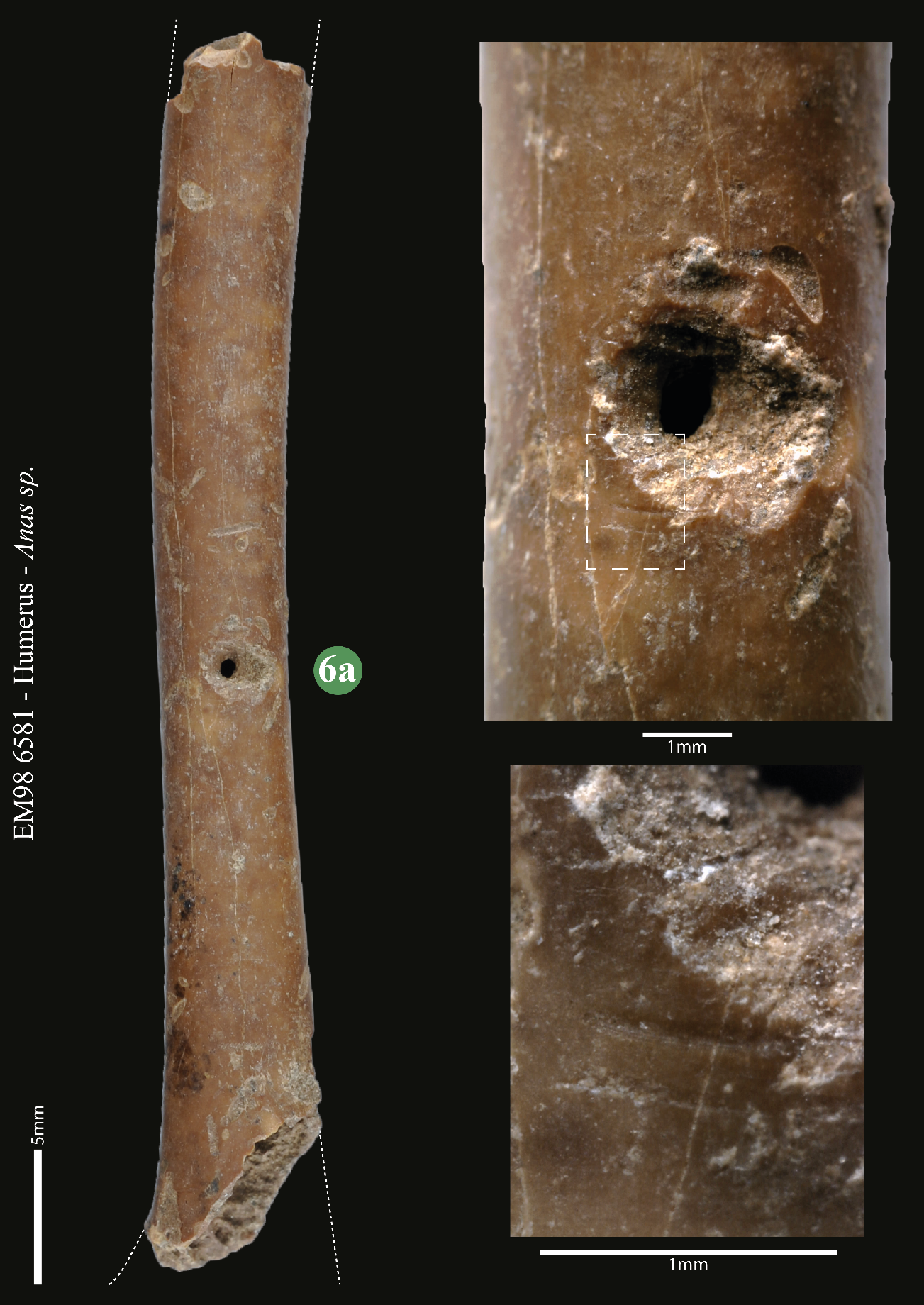


**Fig. S6.** Detail pictures (50-250x) of the technical traces (in green perforation) on the fragmented aerophone (EM98 6581) made of a left humerus of *Anas sp.* (CAD & photos L.D.).


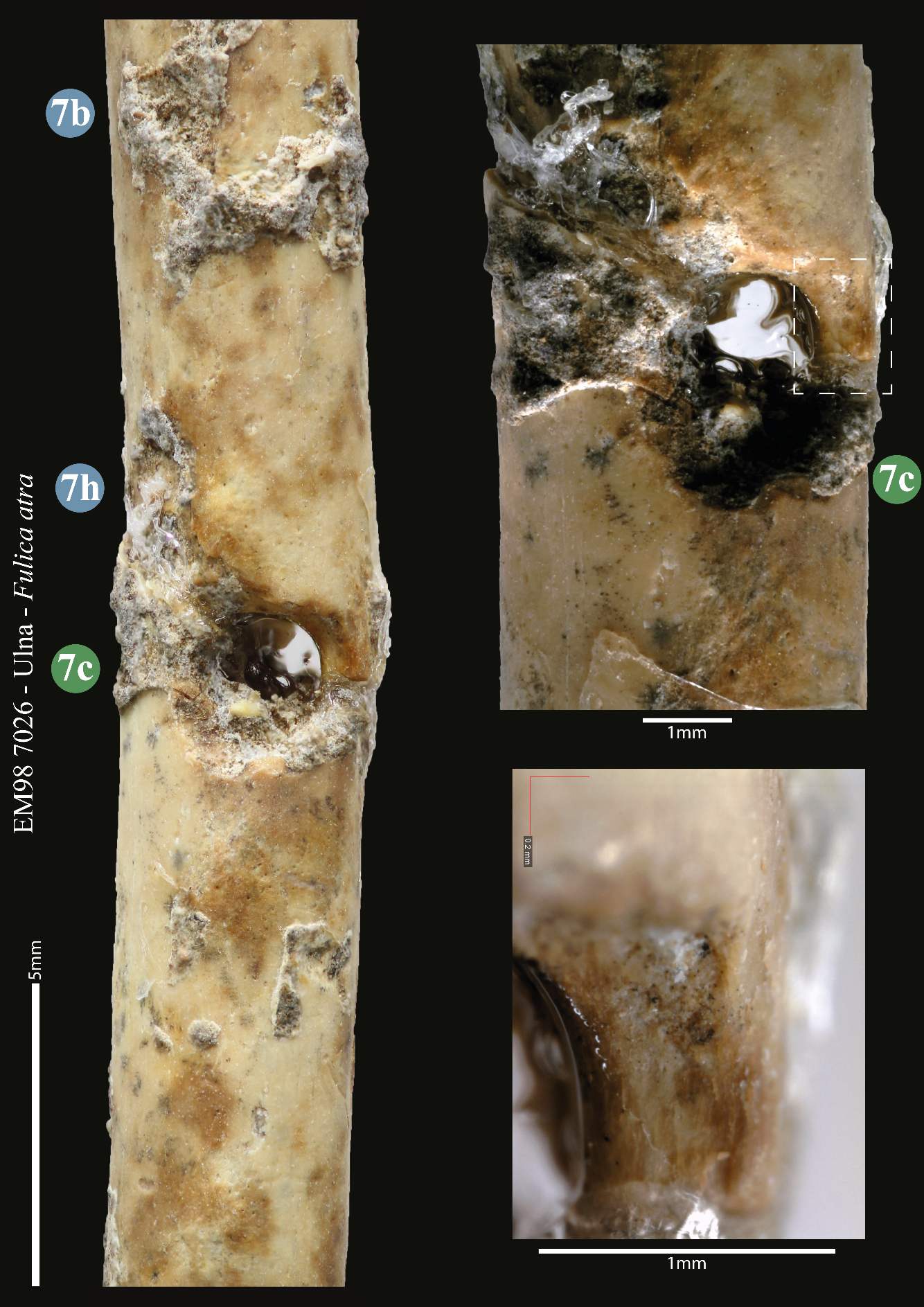


**Fig. S7.** Detail pictures (50-250x) of the technical traces (perforation 7c, markings 7b and 7h) on the complete aerophone (EM98 7026) made of a left ulna of Eurasian coot (*Fulica atra*). (CAD & photos L.D.).


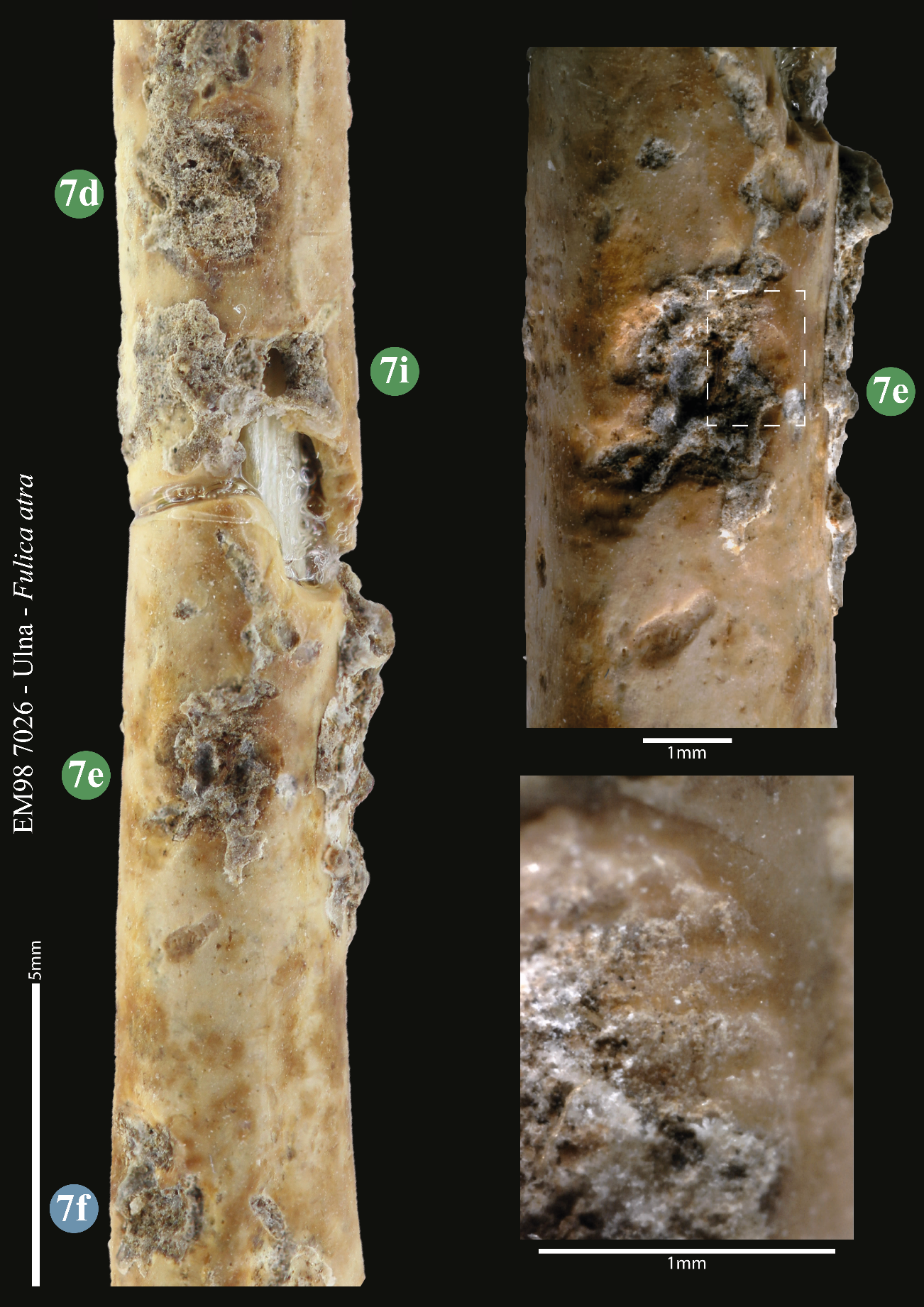


**Fig. S8.** Detail pictures (50-250x) of the technical traces (perforations 7d, 7e, 7i ; marking 7f) on the complete aerophone (EM98 7026) made of a left ulna of Eurasian coot (*Fulica atra*). (CAD & photos L.D.).


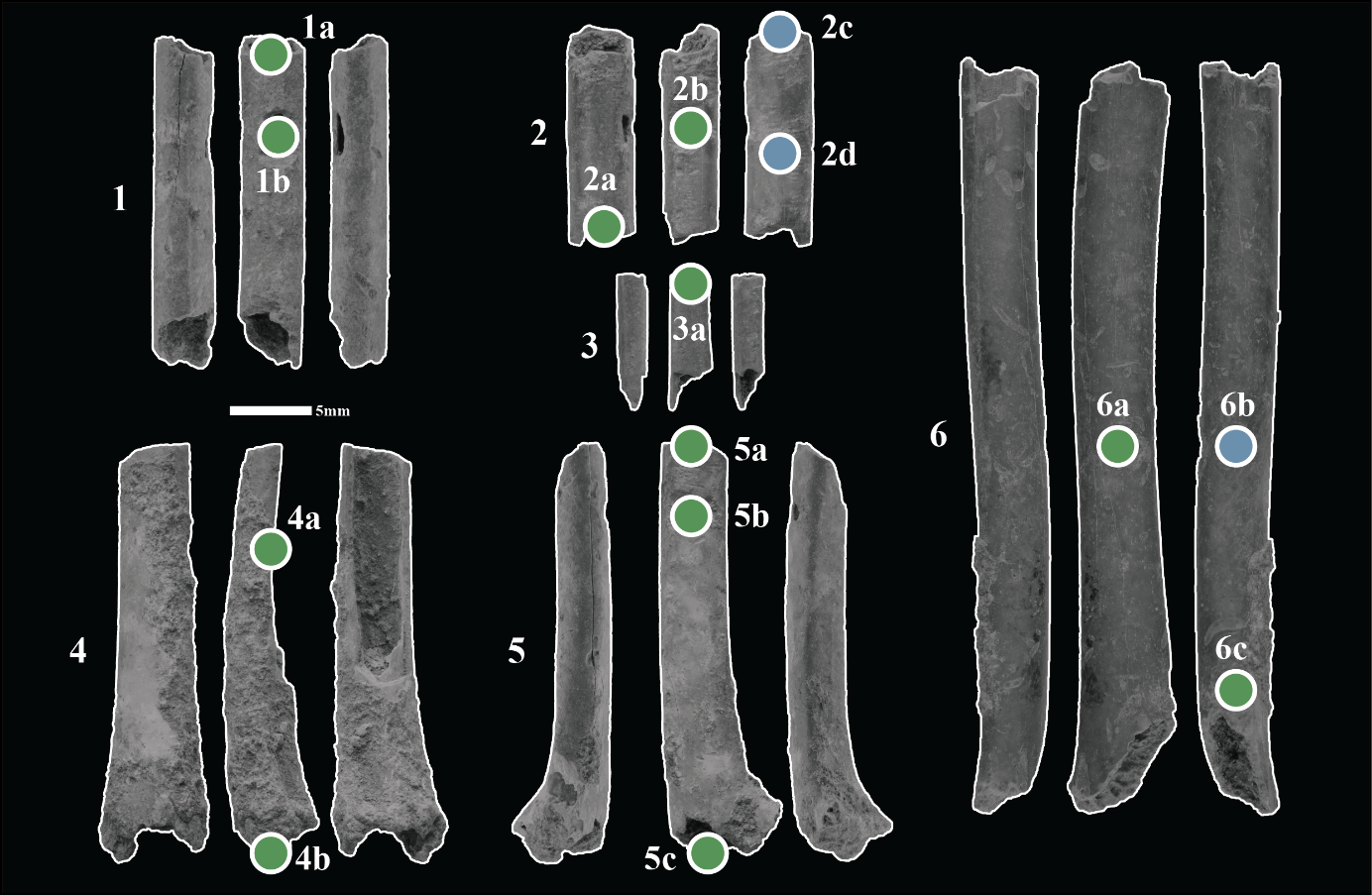


Fig. S9. Detail of the position of the 15 worked areas on the 6 fragmented bone aerophones from Eynan-Mallaha, level Ib (Final Natufian) (in green perforations, in blue markings). 1: EM98 7026; 2: EM96 5564; 3: EM97 6182; 4: EM99 7414; 5: EM04 9363; 6: EM98 6581. (CAD & photos L.D.).

**
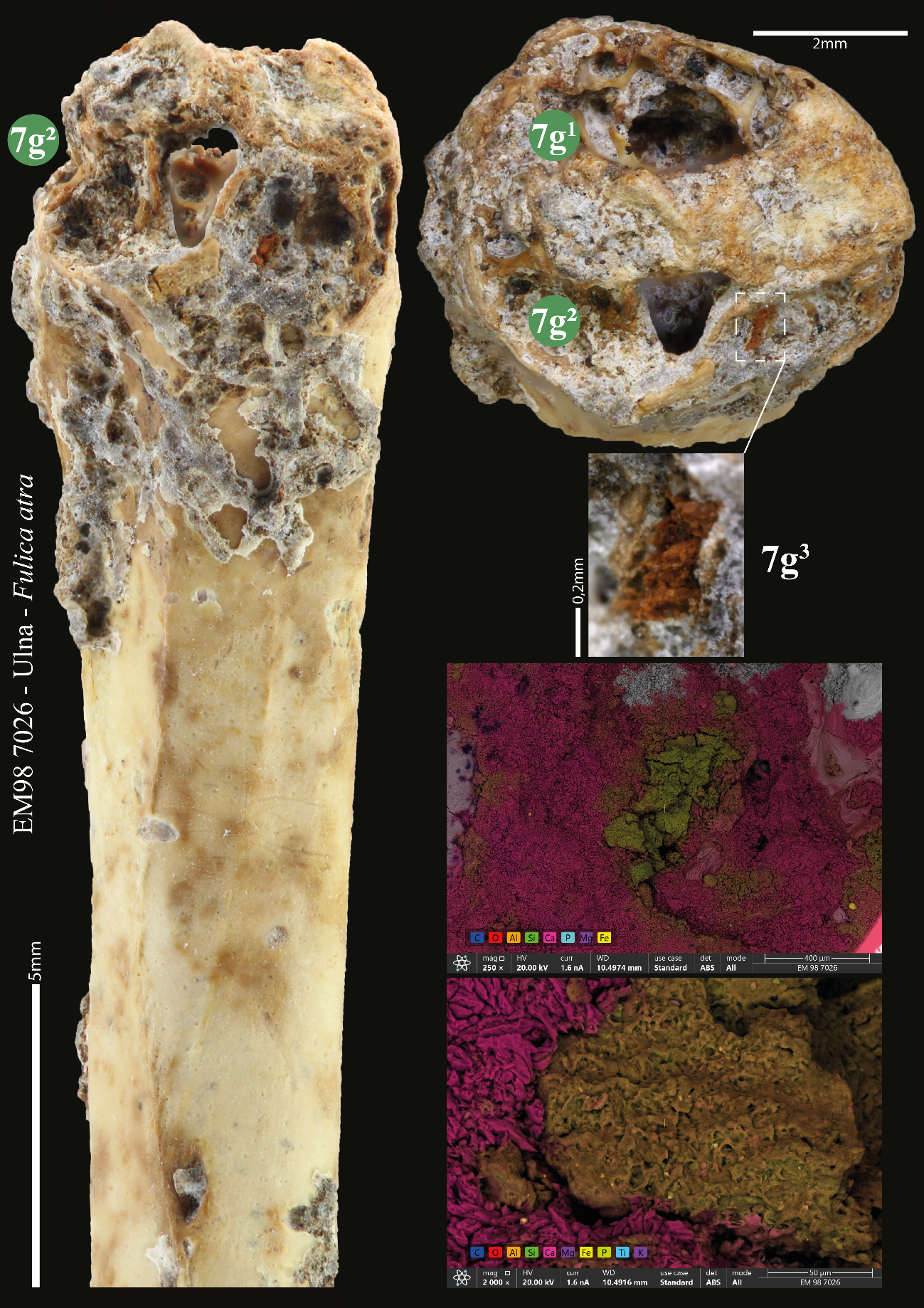
**

**Fig. S10.** Detail pictures (50-250x) of the technical traces (mouthpiece perforations 7g^1^ and 7g^2^) on the complete aerophone (EM98 7026) and the red colourant matter (7g^3^) identified by SEM-EDS as clay and iron oxide (hematite). (CAD & photos L.D.).

**
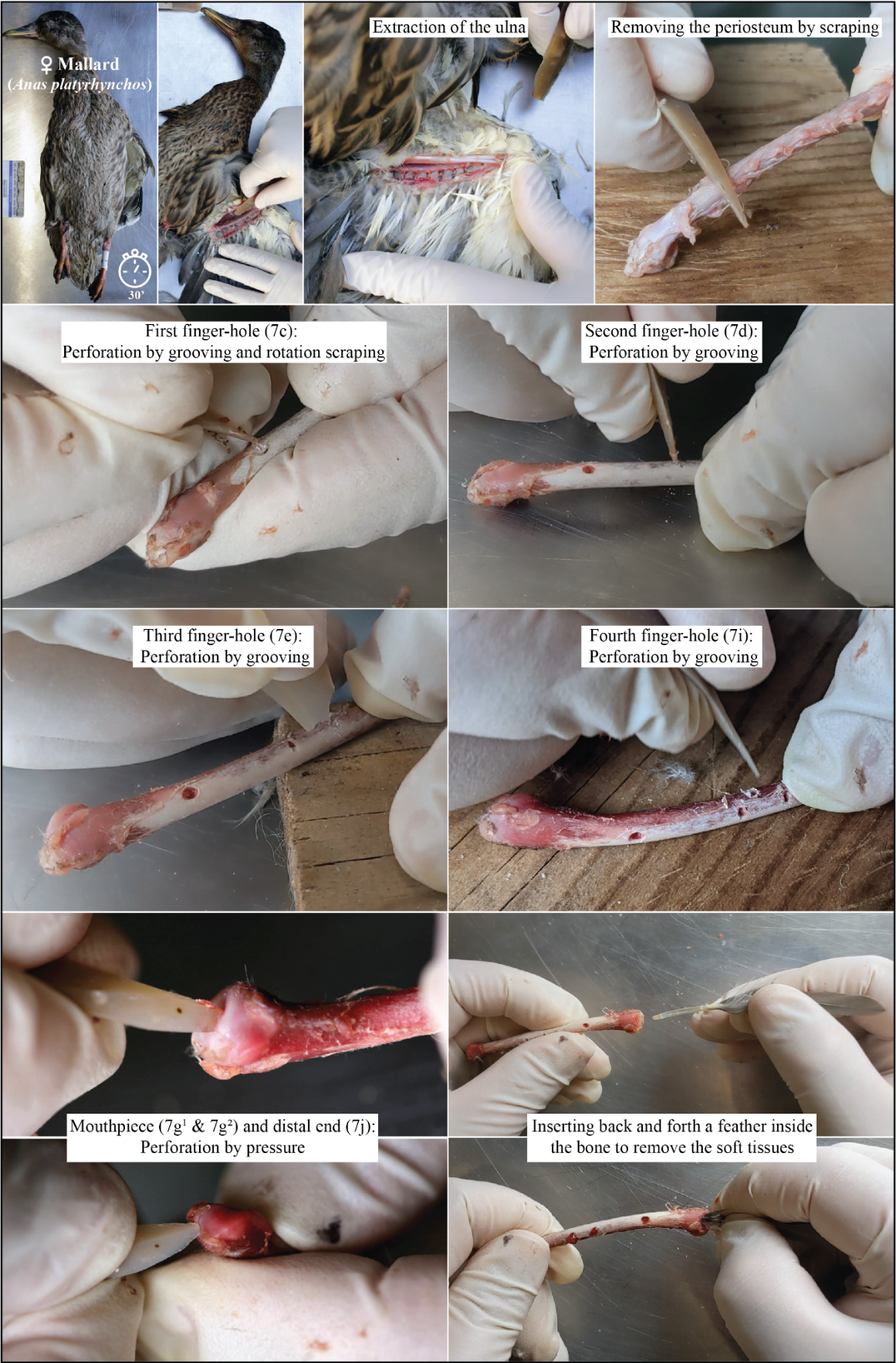
**

**Fig. S11.** Experimental manufacture of the replica of the complete aerophone of Eynan-Mallaha (EM98 7026) made with a green ulna of a female mallard. (CAD & photos L.D.).


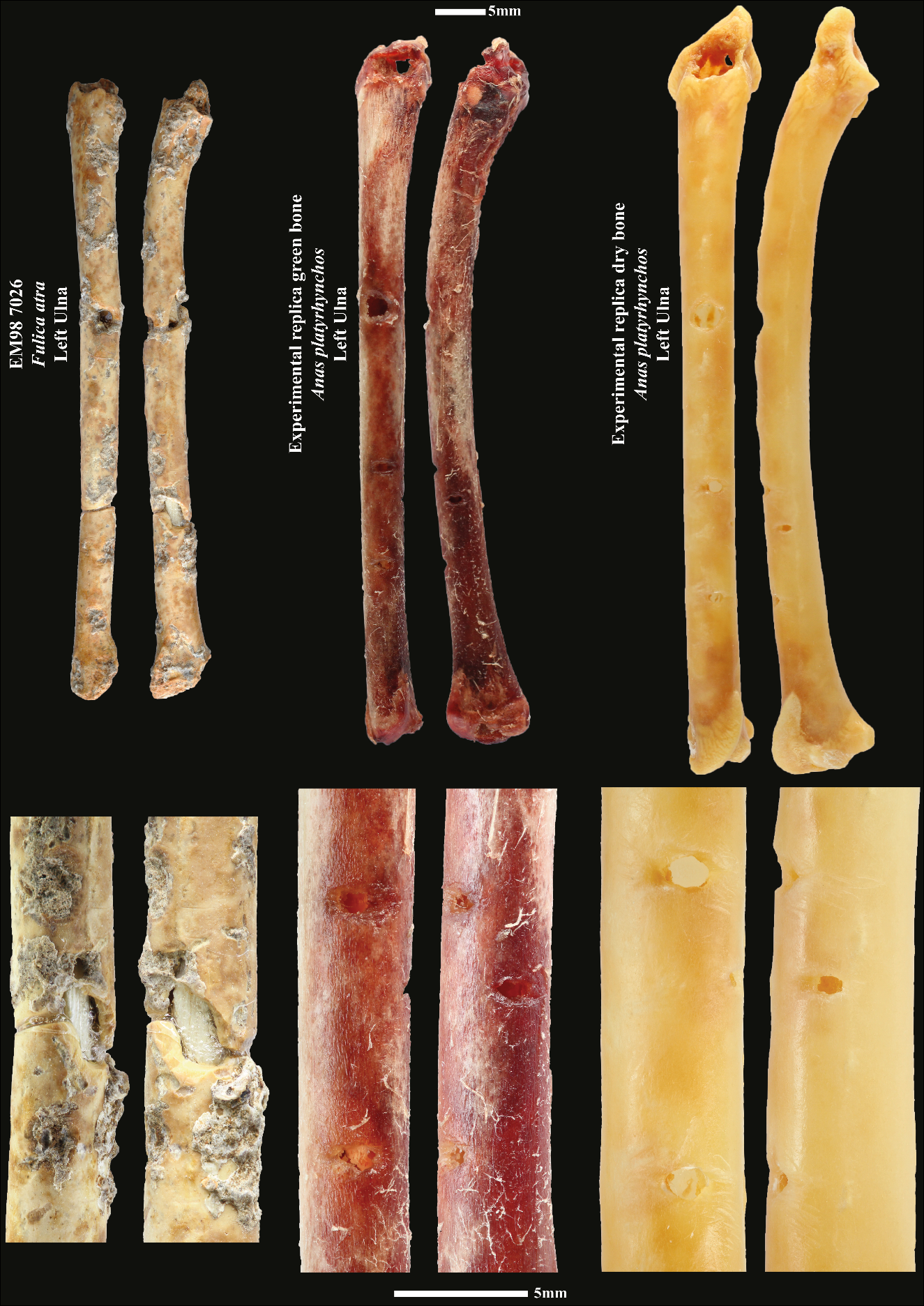


Fig. S12. Comparison of the complete aerophone of Eynan-Mallaha (EM98 7026) with the experimental replicas made on a female mallard’s green and dry left ulna. (CAD & photos L.D.).

**
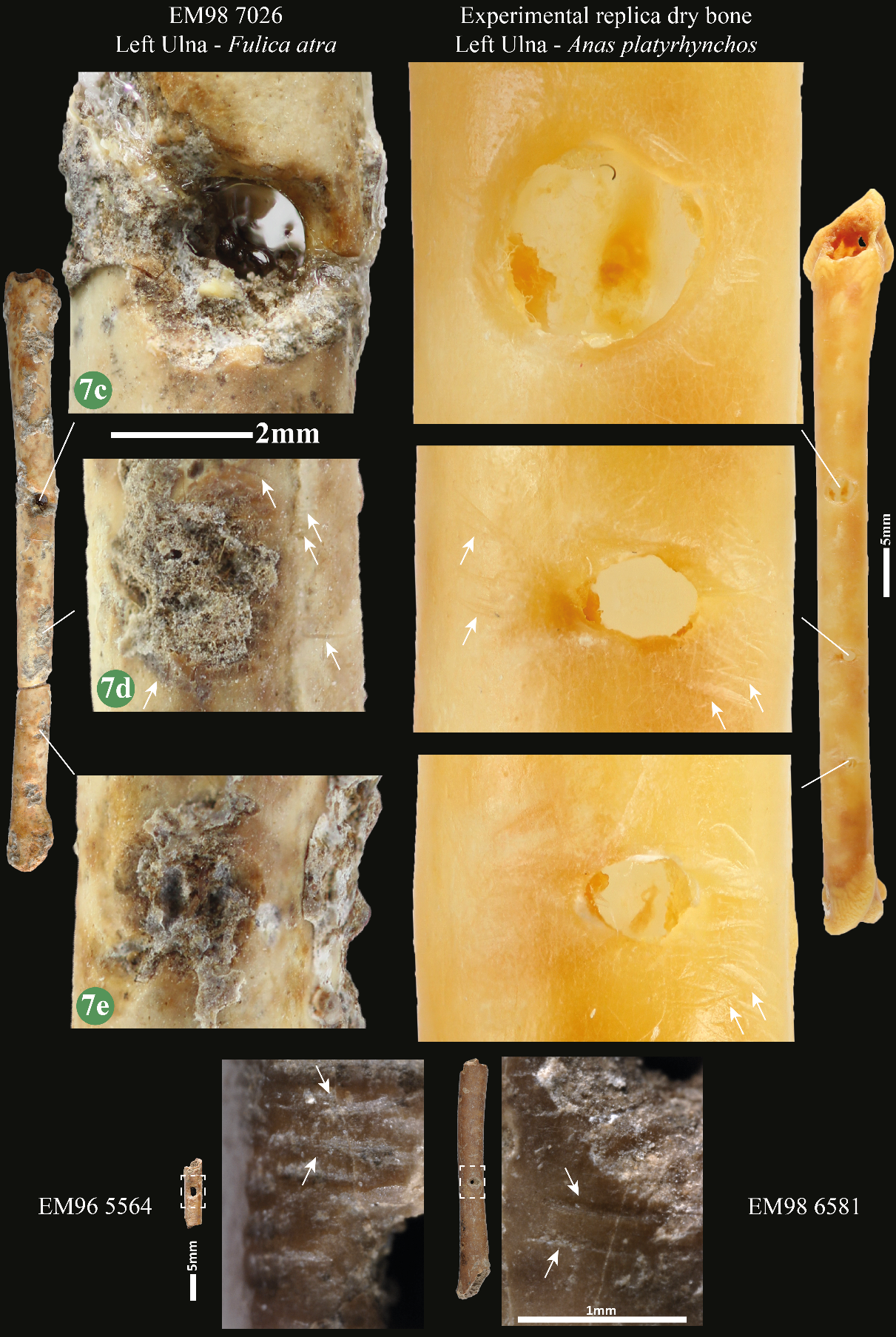
**

Fig. S13. Detail comparison of the technical traces on three aerophones of Eynan-Mallaha (EM98 7026; EM96 5564; EM98 6581) with the experimental replica. The arrows indicate the skid marks of the flint on the bone. (CAD & photos L.D.).


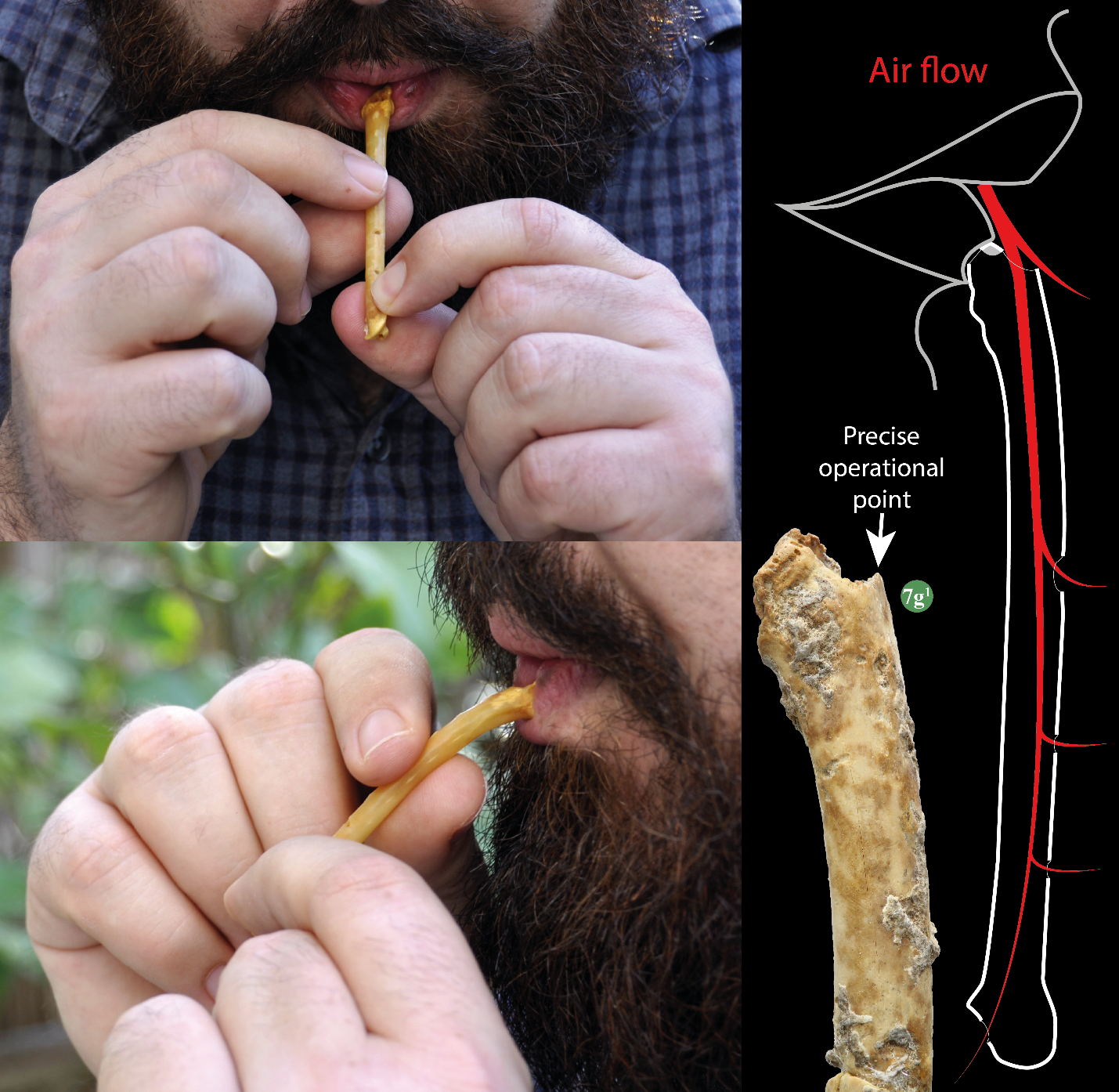


**Fig. S14.** Left: Picture of LD playing the experimental replica of the complete aerophone (EM98 7026) to show the position of the mouthpiece on the lower lip and the fingers on the play-holes.

Right: Schematic illustration of the air flow in the complete aerophone (EM98 7026). The air flow is brought in front of the edge of the mouthpiece perforation which forms the bottom of a notch. The player must hold the instrument against his lips so that the air flow reaches the precise operational point. (CAD & photos L.D.).

**
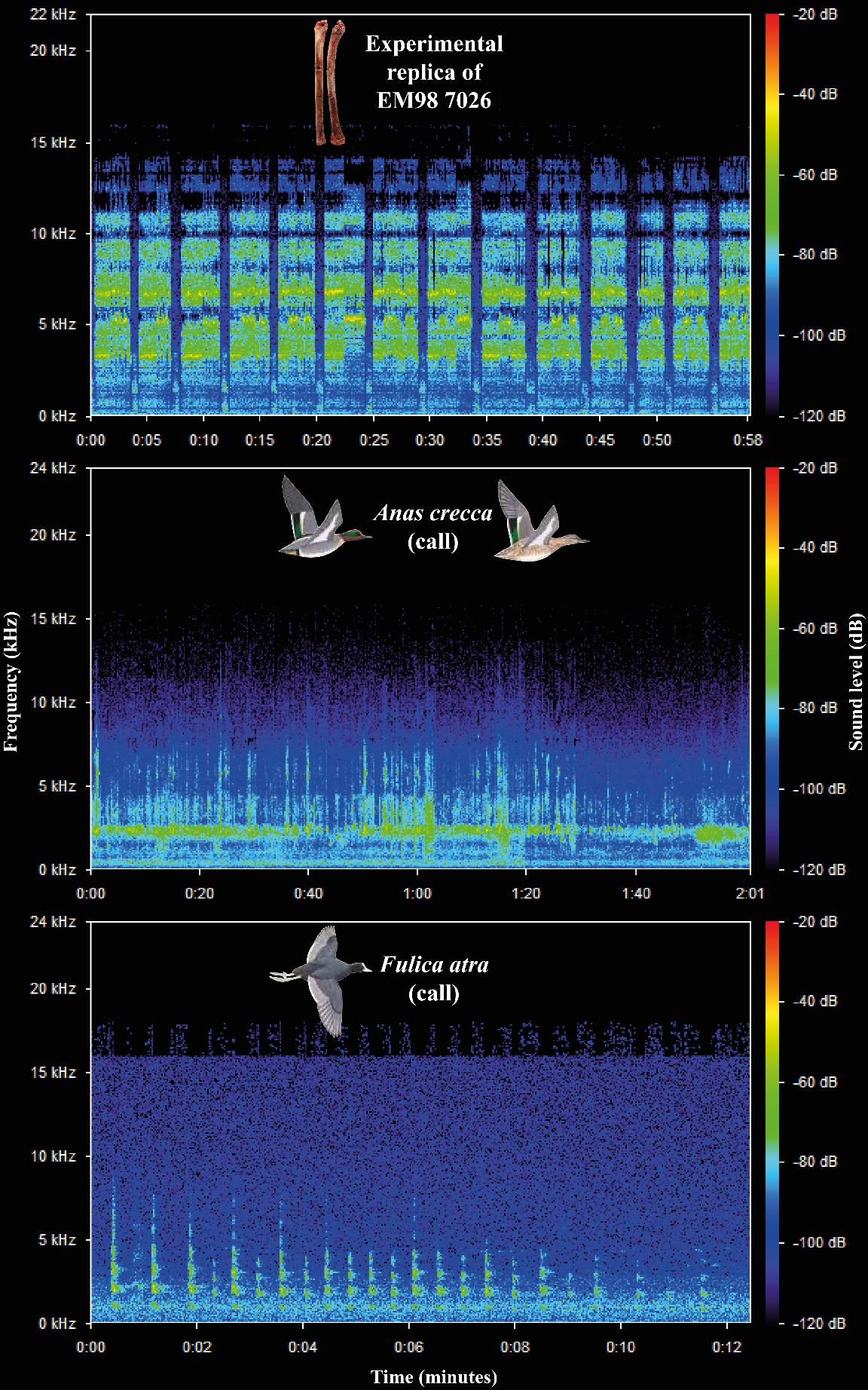
**

**Fig. S15.** Sonogram of the sounds produced by the green bone experimental replica of the complete aerophone EM98 7026 (based on Audio S1); The Eurasian teal (*Anas crecca*) call (based on Audio S2); (D) The Eurasian coot (*Fulica atra*) call (based on Audio S5). (CAD L.D.).

**
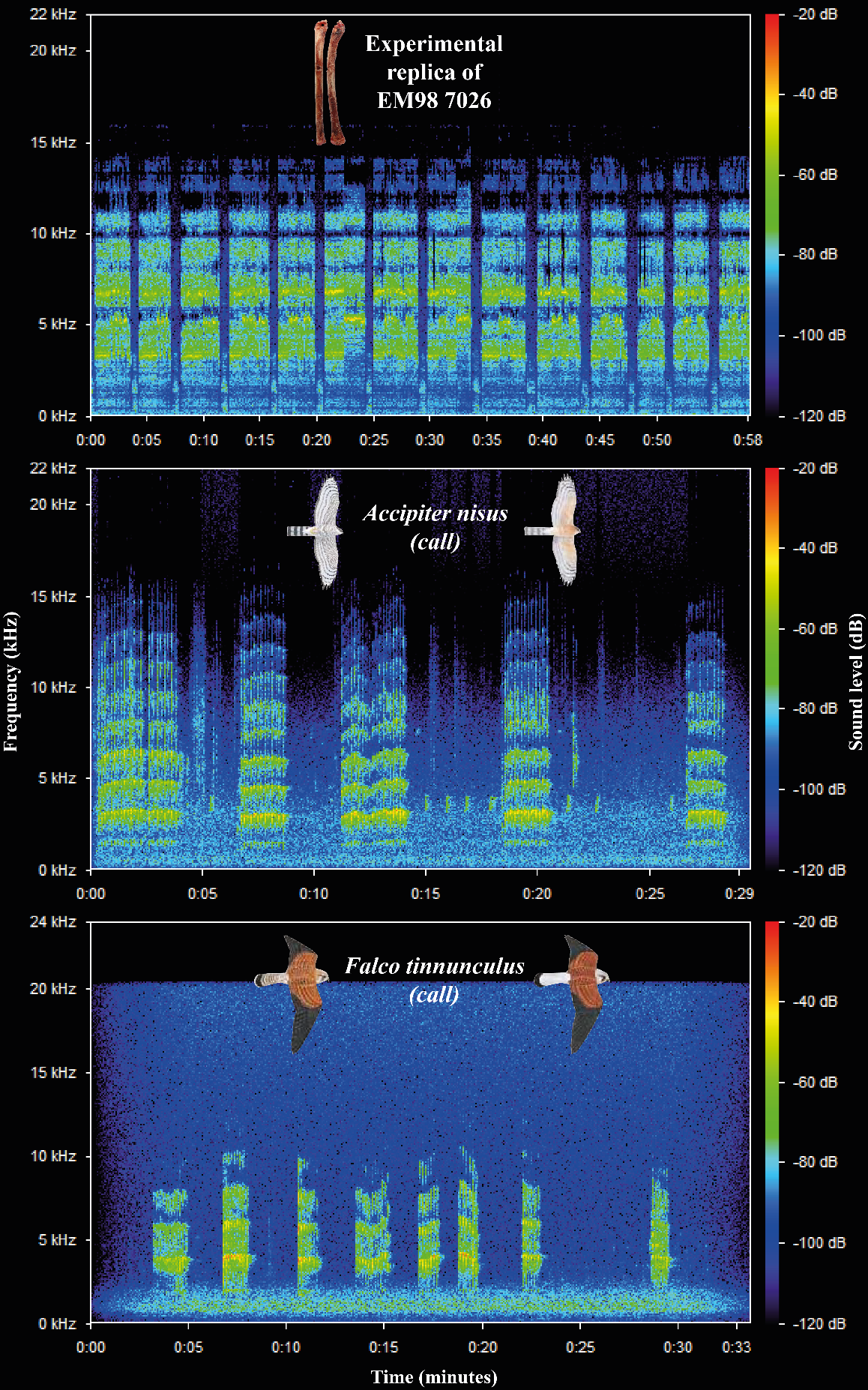
**

**Fig. S16.** Sonogram of the sounds produced by the green bone experimental replica of the complete aerophone EM98 7026 (based on Audio S1); The Sparrowhawk (*Accipiter nisus*) call (based on Audio S4); The Common kestrel (*Falco tinnunculus*) call based on Audio S3). (CAD L.D.).

**
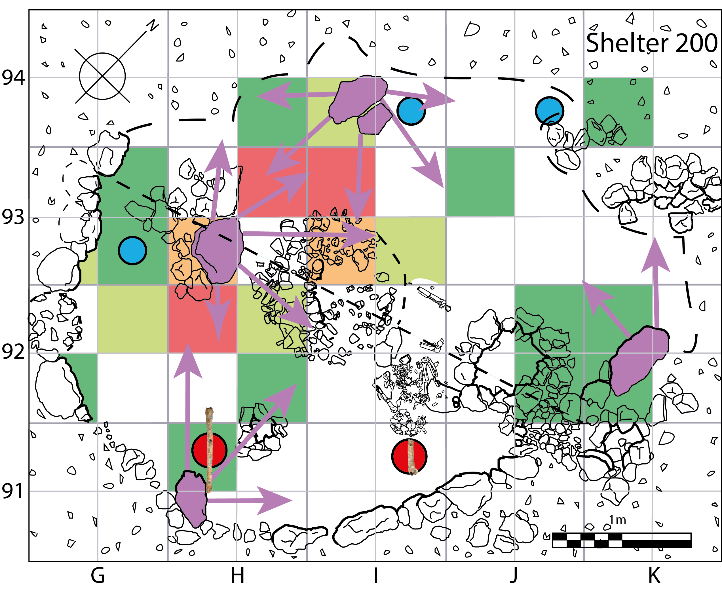

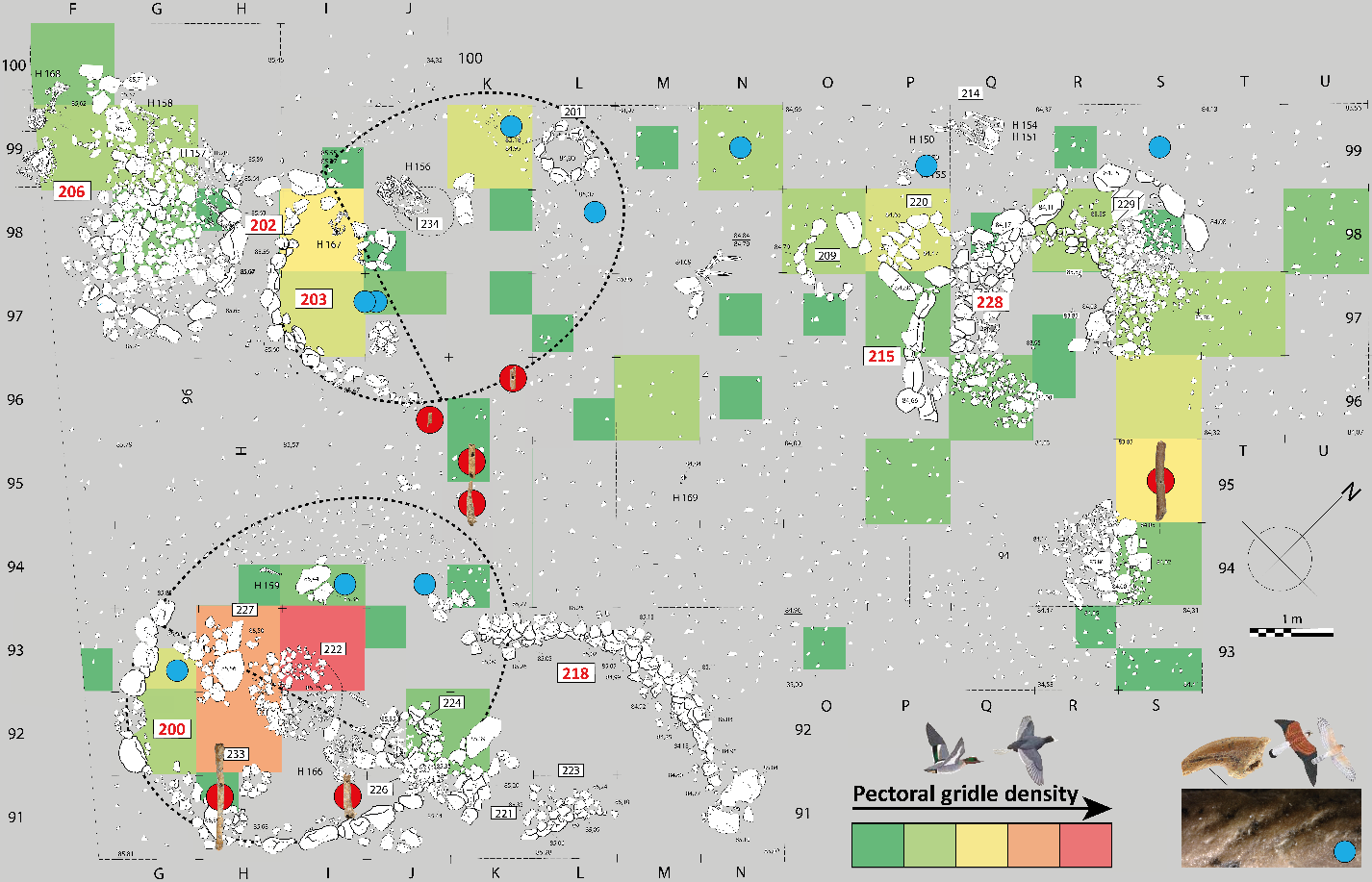
**

**Fig.** **S17**. Top: Plan of the level Ib (Final Natufian) at Eynan-Mallaha (shelters numbers written in red, boundaries indicated for shelters 200 and 203 (the south delimited part is the roofed part) with: the position of the 7 bone aerophones indicated by red dots; the spatial distribution of the pectoral girdle bones (scapula, furculum, sternum and coracoid which supports the flight muscles and is associated with the largest meat mass in the avian body) of Eurasian teal (*Anas crecca*) and Eurasian coot (*Fulica atra*) (NISP=128) ; the position of terminal pedal phalanges (talons) of Common kestrel (*Falco tinnunculus*) and Sparrowhawk (*Accipiter nisus*) indicated by blue dots (NISP=10).

Bottom: Detail (by sub squares) of Shelter 200 with the representation of the artefact distribution from the identified activity stations (in purple)^14^. (CAD L.D.).

**
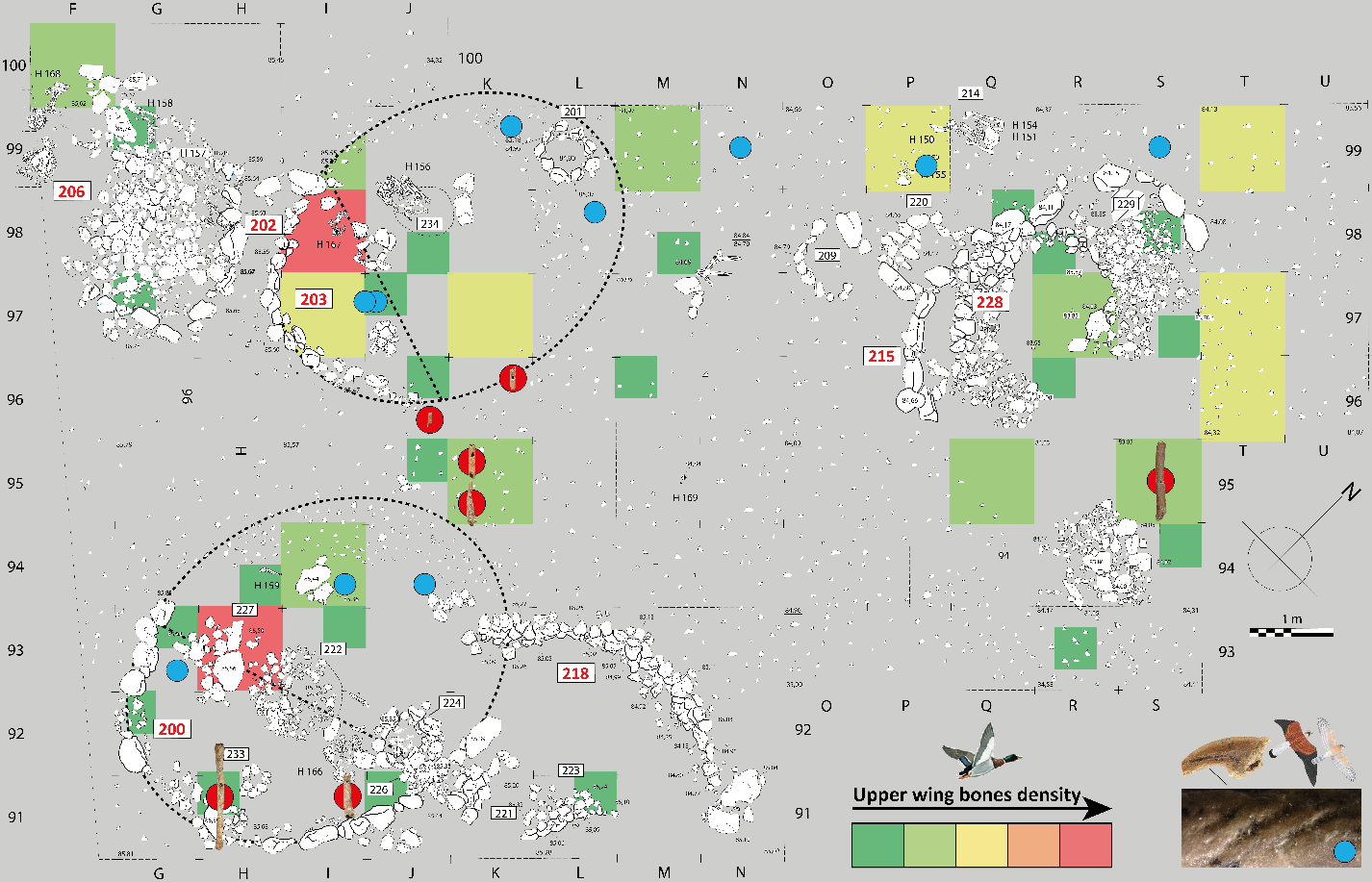
**

**Fig. S18.** Plan of the level Ib (Final Natufian) at Eynan-Mallaha (shelters numbers written in red, boundaries indicated for shelters 200 and 203 (the south delimited part is the roofed part) with: the position of the 7 bone aerophones indicated by red dots; the spatial distribution of upper wing bones (humerus, ulna, radius) of the Mallard (*Anas platyrhynchos*) (NISP=68) ; the position of terminal pedal phalanges (talons) of Common kestrel (*Falco tinnunculus*) and Sparrowhawk (*Accipiter nisus*) indicated by blue dots (NISP=10). (CAD L.D.).

**Table S1.** List of bird species identified in Eynan-Mallaha, level Ib (Final Natufian). Waterfowl used to make the aerophones in orange and raptors, whose call auditory profile aligns with the aerophone, in yellow. NISP = number of identified specimens.

**Table S2.** Details and measurements of the 26 worked areas on the 7 bone aerophones from Eynan-Mallaha, level Ib (Final Natufian). 14 finger-holes are highlighted in green, 3 distal end perforations in grey, and the 7 markings in blue.

Audio S1.

MP3 Sound recording of the green bone experimental replica of the complete aerophone (EM98 7026) from Eynan-Mallaha. Player and Recorder: L. Davin.

(MP3 is provided as separate file)

Audio S2.

MP3 Sound recording of Eurasian teal (*Anas crecca*) (MNHN-SO-2022-13).

(MP3 is provided as separate file)

Audio S3.

MP3 Sound recording of Common kestrel (*Falco tinnunculus*) (XC758230).

(MP3 is provided as separate file)

Audio S4.

MP3 Sound recording of Sparrowhawk (*Accipiter nisus*) (XC736470).

(MP3 is provided as separate file)

Audio S5.

MP3 Sound recording of Eurasian coot (*Fulica atra*) (XC737791).

(MP3 is provided as separate file)
